# Postmarketing Assessment of Antibody–Drug Conjugates: Proof‐of‐Concept Using Model‐Based Meta‐Analysis and a Clinical Utility Index Approach

**DOI:** 10.1002/psp4.70013

**Published:** 2025-03-04

**Authors:** Innocent Gerald Asiimwe, Nour Chtiba, Samer Mouksassi, Goonaseelan (Colin) Pillai, Raimund M. Peter, Eunice Yuen, Venkatesh Pilla Reddy

**Affiliations:** ^1^ The Wolfson Centre for Personalized Medicine, Department of Pharmacology and Therapeutics Institute of Systems, Molecular and Integrative Biology, University of Liverpool Liverpool UK; ^2^ APT‐Africa Fellowship Program, c/o Pharmacometrics Africa NPC Groote Schuur Hospital Cape Town South Africa; ^3^ Pharmacy of Monastir University of Monastir Monastir Tunisia; ^4^ Certara Cairo Egypt; ^5^ Division of Clinical Pharmacology University of Cape Town South Africa; ^6^ CP+ Associates GmbH Basel Switzerland; ^7^ Global PKPD/Pharmacometrics, Eli Lilly and Company Bracknell UK

**Keywords:** antibody–drug conjugates, clinical utility index, model‐based meta‐analysis, trastuzumab deruxtecan, trastuzumab emtansine

## Abstract

Antibody–drug conjugates (ADCs) are a promising class of targeted cancer therapies. However, they need careful dose optimization to maximize effectiveness and minimize side effects. Sometimes, safety issues may only become apparent after approval, so ongoing evaluation is important. This study aimed to assess the benefit–risk profiles of two approved trastuzumab–drug conjugates: trastuzumab emtansine (T‐DM1) and trastuzumab deruxtecan (T‐DXd). A systematic search of MEDLINE on May 1, 2024, identified clinical trials reporting the pharmacokinetics, pharmacodynamics, safety, and efficacy of T‐DM1 and T‐DXd. Summary‐level data from 103 trials was used along with model‐based meta‐analysis to develop population pharmacokinetic and exposure–response models for both ADCs. The study combined the objective response rate (ORR) and dose‐limiting toxicity (DLT) into a composite score called the clinical utility index (CUI) to determine optimal drug exposures and doses that maximize the benefit–risk balance. Different ORR/DLT weights and CUI thresholds representing desired minimum effect size were tested in three scenarios (“only phase I trials,” “phase I/II trials,” and “all phases”). Using a CUI threshold of 10%, the approved T‐DM1 dose of 3.6 mg/kg for breast cancer was found to align with an efficacy–safety (ORR‐DLT) ratio of 81:19 with all phases. Applying these weights to the T‐DXd analyses successfully predicted the approved T‐DXd dose (5.4 mg/kg, breast cancer), showing a CUI improvement compared to the 3.2 mg/kg dose of 8.3%, 8.2%, and 2.4% in the three respective scenarios. Overall, this proof‐of‐concept assessment of ADCs can save time and costs for pharmaceutical companies and optimize dosing to maximize patient benefit.

**Summary**

- What is the current knowledge on the topic?
  ○ Antibody–drug conjugates (ADCs) are a promising class of targeted cancer therapies that require careful dose optimization to maximize effectiveness and minimize side effects.
- What question did this study address?
  ○ Can a model‐based meta‐analysis (MBMA) and a clinical utility index (CUI) approach be used to assess the benefit–risk profiles of ADCs?
- What does this study add to our knowledge?
  ○ Using two approved trastuzumab–drug conjugates, trastuzumab emtansine (T‐DM1) and trastuzumab deruxtecan (T‐DXd), and three scenarios involving only Phase I trials, Phase I/II trials, and all phases/trials, respectively, we have demonstrated how MBMA, supplemented by a systematic review, and a CUI approach that combines efficacy and safety outcomes into a composite score, can be used to assess the benefit–risk profiles of ADCs.
- How might this change drug discovery, development, and/or therapeutics?
  ○ This proof‐of‐concept assessment of ADCs can save time and costs for drug developers and optimize dosing to maximize patient benefit. Although demonstrated with ADCs, this approach is broadly applicable to other drug modalities.

## 1 Introduction

Antibody–drug conjugates (ADCs) represent a promising class of targeted cancer treatments. They combine the precision of monoclonal antibodies with the potent cytotoxicity of small‐molecule drugs [1]. ADCs are designed to deliver drugs exclusively to target cancerous cells, sparing healthy tissue cells. This thereby maximizes efficacy, minimizes systemic side effects, and, compared to the small molecules alone, widens the therapeutic window [2]. Anti‐human epidermal growth factor receptor 2 (HER2) therapies focus on targeting HER2‐expressing cancer cells—the pioneering monoclonal antibody in this field is trastuzumab. Trastuzumab drug conjugates (T‐DCs) leverage trastuzumab's specificity for cancer cells to deliver cytotoxic drugs directly into them. Notable T‐DCs include trastuzumab‐emtansine (T‐DM1), which is approved for breast cancer, and trastuzumab deruxtecan (T‐DXd), approved for breast, lung, and other cancers. These received initial US FDA approvals in 2013 and 2019, respectively [1, 3].

The success of ADCs in clinical settings relies on finding the right balance between effectiveness and safety. This balance involves carefully evaluating key factors such as the target antigen (the molecule on cancer cells that the ADC targets), antibody (which guides the drug to the cancer cells), linker (which connects the antibody to the drug), conjugation technology (the method of attaching the drug to the antibody), and payload (the cytotoxic drug delivered to the cancer cells). It is crucial to select the proper dosage regimen to maximize effectiveness and minimize adverse effects. Evaluating the benefits versus the risks of drugs, including ADCs, is an ongoing process. A study in the United States emphasized the importance of continuous safety evaluation. It revealed that 21% of FDA‐approved new molecular entities across various therapeutic areas from 1980 to 1999 required dose adjustments, primarily due to safety issues identified postapproval [4]. The FDA's guidance for the industry, “Benefit‐Risk Assessment for New Drug and Biological Products,” recognizes that a drug's benefits and risks can change over time and recommends continuous benefit–risk assessments throughout the drug's lifecycle [5]. Additionally, the FDA and American Association for Cancer Research strongly encourage a holistic approach that utilizes all existing evidence during benefit–risk assessments [6].

Model‐based meta‐analysis (MBMA) is a quantitative technique that integrates summary data from published clinical trials and potentially internal data to support critical drug development decisions, including benefit–risk assessments of investigational or approved treatments [7]. Clinical utility index (CUI) is a multiattribute decision analysis tool used to quantitatively assess the benefits and risks of a product, understand its therapeutic index, and evaluate its competitive advantage [8, 9, 10]. The CUI makes use of multiple efficacy and safety attributes and has been previously utilized to guide dose selection during drug development based on early trial (Phase I or II) results, as well as to personalize the doses for an approved drug [8, 9, 10, 11]. The FDA promotes the integration of a drug's benefits and risks into a combined analysis for conducting a benefit–risk assessment [5]. During benefit–risk assessments, exposure‐based therapeutic index calculations, which relate a drug's pharmacokinetic (PK) exposure to its clinical efficacies and adverse effects, may be preferable to dose‐based approaches due to variability in patient PK exposure for the same dose [12]. This approach can also enhance understanding of second‐generation ADCs and contribute to improvements in therapeutic indices [12].

The aim of this analysis was to evaluate the benefit–risk profiles of two T‐DCs: trastuzumab emtansine (T‐DM1) and trastuzumab deruxtecan (T‐DXd). T‐DM1 is administered at a dose of 3.6 mg/kg every 3 weeks (21‐day cycle) for breast cancer [13]. T‐DXd, also administered in a 21‐day cycle, is given at a dose of 5.4 mg/kg for indications such as breast cancer and non–small‐cell lung cancer, or 6.4 mg/kg for indications such as gastric cancer [14]. Both T‐DCs are administered as intravenous infusions, with the duration being 90 min in Cycle 1 and 30 min in subsequent cycles if the drugs are well tolerated. We used MBMA and summary‐level data from clinical trials to develop population‐PK (Pop‐PK) and exposure–response models for the two T‐DCs and combined key safety and efficacy data into a CUI. The CUI was then used to determine drug exposures and doses that achieve the optimal balance between benefits and risks. We simulated different stages of drug development by considering “post‐approval” scenarios, which included all identified studies, and “pre‐phase III” scenarios, which only included studies conducted before Phase III.

## 2 Methods

### 2.1 Systematic Review of the Literature to Identify Clinical Trials

To identify clinical trials for our analysis, we conducted a systematic search of MEDLINE through Ovid (https://ospguides.ovid.com/OSPguides/medline.htm) on May 1, 2024, using search terms related to trastuzumab, emtansine, deruxtecan, pharmacokinetics, pharmacodynamics, efficacy, and safety. Additionally, we reviewed reference lists from the identified studies, previous systematic reviews, and other relevant sources to identify further eligible trials. Detailed information on the search strategy, additional sources, study selection criteria, and the data extraction process can be found in Text S1.

### 2.2 Population Pharmacokinetic Model Development Using Model‐Based Meta‐Analysis (MBMA)

We used summary‐level data from clinical trials and nonlinear mixed‐effects modeling in Monolix (version 2024R1, https://monolix.lixoft.com/) via R (version 4.4.1) [15] with the package lixoftConnectors [16] to develop population pharmacokinetic (Pop‐PK) models for both ADCs. We extracted data from only the first dose (Cycle 1). Given the elimination half‐lives of approximately 4 days for T‐DM1 and 6 days for T‐DXd, [17] Cycle 1 can effectively represent steady‐state conditions, and no accumulation was observed for these ADC drugs when given in 21‐day cycles [18]. Pop‐PK input datasets were programmed in R, with missing body weight data imputed using the median body weight from other studies, adjusted for the number of subjects per study (see https://github.com/iasiimwe/adc_cui for details). Based on prior literature, [19, 20, 21] we implemented two‐compartment models with linear elimination and also explored nonlinear clearance/target‐mediated disposition [20, 22]. We parameterized the structural model using clearances and distribution volumes. An exponential random effects term was used to model between‐study variability (BSV) in the parameters, while proportional effects were used to model residual error, with residual error weighted by the square root of a trial's sample size [23, 24]. We treated different doses within the same study as separate studies rather than trial arms, thus accounting for between‐treatment arm variability (BTAV) under BSV. BSV represents differences in inclusion criteria across studies, whereas BTAV arises when patients recruited using the same criteria are split into two or more arms [23, 24]. In early‐phase studies, such as Phase I dose escalation studies, the same population is not randomized, but rather patients may be recruited sequentially based on dose. Therefore, it may be more accurate to model different arms/doses as different studies (i.e., model BSV instead of BTAV). However, in sensitivity analysis, we evaluated the effect of modeling BTAV separately from BSV and assessed the impact of including only “dose‐finding” studies, defined as Phase 1 trials reporting data for at least two dose levels. We evaluated the fitted models using a combination of graphical analysis (goodness‐of‐fit plots and visual predictive checks), parameter estimate precision (we aimed for relative standard errors of < 30% for fixed effect parameter estimates and < 50% for random effect parameter estimates), and the ability to match the observed concentrations (see section “Bayesian Individual Dynamic Predictions with Uncertainty”).

### 2.3 Bayesian Individual Dynamic Predictions With Uncertainty

We used Monolix and Simulx (version 2024R1) via R (version 4.4.1) with the package lixoftConnectors to simulate Bayesian individual dynamic predictions with uncertainty [15, 25]. Briefly, using the distribution of parameters of the structural model as priors, Conditional distribution sampling was performed in Monolix to obtain posterior distributions using Markov chain Monte Carlo procedures, which were then used for model simulations with Simulx. One thousand replicates were performed for each dose level/trial (7 dose levels or 7000 subjects in total for each ADC), with the observed median weight across studies used when computing the T‐DM1/T‐DXd amount.

### 2.4 Exposure–Response (ER) Analysis

The efficacy endpoints included the proportion of participants who survived or were censored during follow‐up (overall survival), did not experience disease progression (progression‐free survival), and achieved a response (objective response rate).

We focused on dose‐limiting toxicity (DLT) as the safety endpoint, defined as the most frequent occurrence of DLT (if reported by the primary study), dose reduction, drug discontinuation, or drug interruption/delay. Only DLTs attributed to drug toxicity were included when reasons were provided. Text S2 details the rationale for choosing DLT over alternatives (based on T‐DM1/T‐DXd data), such as serious adverse events, and explains the method for deriving this composite endpoint.

We considered three model‐predicted PK exposure metrics in Cycle 1: maximum drug concentrations (Cmax), minimum drug concentrations (Cmin, at 504 h), and the area under the concentration–time curve (AUC) from Time 0 to Day 21 (504 h). AUC_inf (AUC extrapolated to infinity) was also extracted and used to impute missing AUC values using the formula AUC = AUC_inf × AUC ratio, with the ratio (AUC/AUC_inf) derived from studies reporting both metrics. Other missing PK exposures were imputed using the median exposures from other dose levels.

Logistic regression was performed in R using the stats package (glm function, family “quasibinomial,” https://www.rdocumentation.org/packages/stats/versions/3.6.2/topics/family) [15]. PK exposure metrics were used as independent variables, and efficacy/safety endpoints as continuous outcomes, with regression weighted by the square root of the sample size. Each analysis unit was defined by cancer type (if reported separately) and dosing arm, with each cancer‐dose combination analyzed individually (e.g., a study with two cancer types and two dosing arms counted as four units). Unlike Pop‐PK modeling, which included both weekly and three weekly dosing (as PK exposure is consistent across different dosing intervals in the first cycle), the ER analysis focused only on the approved three weekly dosing, as weekly dosing could lead to higher exposures and more outcome events for the same dose. No covariates were included.

### 2.5 Clinical Utility Index (CUI)

**FIGURE 1:**
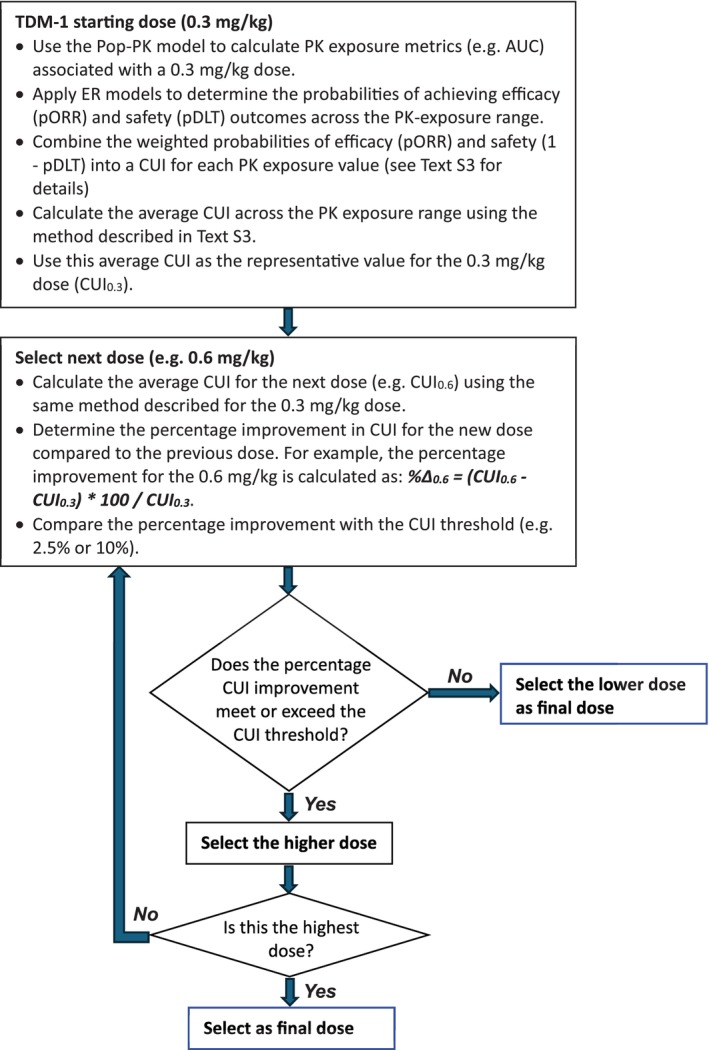
Flowchart for T‐DM1 dose selection using a CUI threshold. Dose selection was guided by testing several CUI thresholds (2.5%–20%), where the threshold represents the minimum percentage improvement in average CUI required to justify selecting a higher dose. For example, for T‐DM1, average CUIs were computed for all dose levels (0.3, 0.6, 1.2, 1.8, 2.4, 3.6, and 4.8 mg/kg), and improvements in CUI between consecutive doses were compared against the threshold. Starting with the lowest dose (0.3 mg/kg), if the CUI improvement to the next dose (0.6 mg/kg) met or exceeded the threshold, the 0.6 mg/kg dose was selected. This comparison continued sequentially for each dose pair (e.g., 0.6 vs. 1.2 mg/kg) until the improvement between two consecutive doses fell below the threshold. The final selected dose was the highest dose where the CUI improvement over the previous dose remained equal to or above the specified threshold, ensuring meaningful dose escalation based on clinical utility. The same process was followed for T‐DXd. Abbreviations: AUC, area under the CUI‐PK exposure curve; CUI, clinical utility index; DLT, dose‐limiting toxicity; ER, exposure response; max, maximum; min, minimum; ORR, objective‐response rate; *p*, probability; PK, pharmacokinetic; Pop‐PK, population pharmacokinetic; T‐DM1, Trastuzumab emtansine; T‐DXd, Trastuzumab deruxtecan.

Key safety and efficacy endpoints were combined into a composite score, the CUI, to determine optimal drug exposures and doses that maximize the benefit–risk balance [8, 9, 10]. The CUI is mathematically defined as a weighted sum of individual utility functions for multiple selected attributes. In its simplest form (additive utility), it is expressed as: *U(x₁, …, x_n_) = ∑wᵢUᵢ(xᵢ)*, where *xᵢ* represents the attribute values, *wᵢ* are the weights summing to 1, and each utility function *Uᵢ(xᵢ)* is scaled between 0 and 1. The process of developing the CUI, including detailed examples, is outlined in Text S3. Weights ranging from 10% to 90% were tested (excluding 0% and 100% as no approved drugs exhibit only unwanted or only beneficial effects). The average CUI per dose was calculated to account for variability in PK exposures associated with a single dose (e.g., intersubject variability), as detailed in Text S3. Several CUI thresholds (2.5%–20%) were evaluated, where the threshold represents the minimum percentage improvement in average CUI required to justify selecting a higher dose. The final selected dose was the highest dose where the CUI improvement over the previous dose met or exceeded the threshold (see Figure 1 for the flowchart). To simulate different stages of drug development, we computed CUIs for three scenarios: “phase I only” (considering only Phase I trials), “phases I and II” (considering both Phase I and Phase II trials), and “all phases” (including all trial phases, from Phase I to Phase III and postapproval/Phase IV). R Shiny was used to explore the effects of changing different components and weights on the average CUI per dose. All code used during analysis is available at https://github.com/iasiimwe/adc_cui.

## 3 Results

### 3.1 Characteristics of Included Studies

**FIGURE 2:**
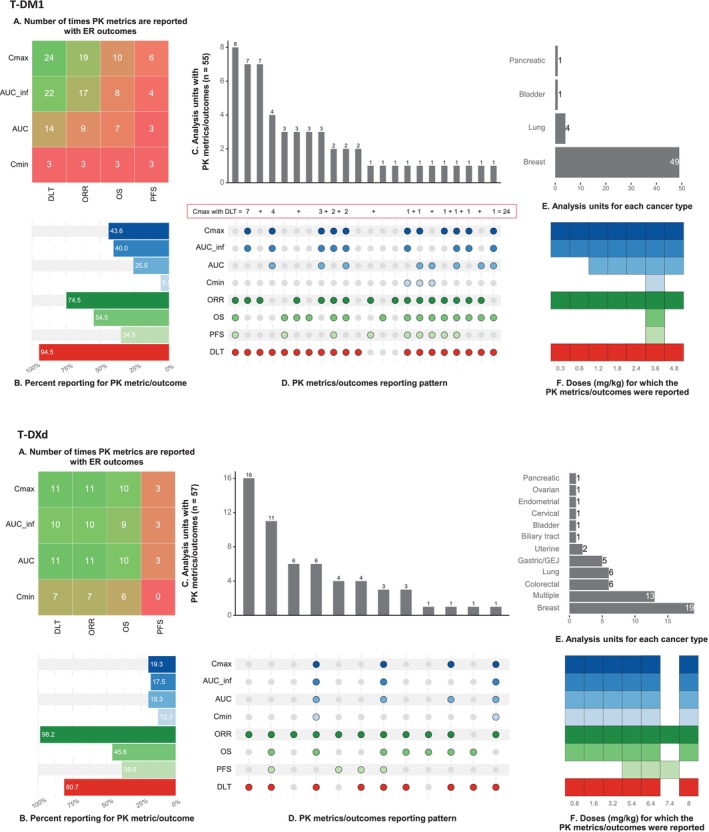
Pharmacokinetic metrics and outcomes included in the exposure–response analyses for T‐DM1 and T‐DXd. Panel A (heat maps) shows the frequency with which PK metrics were reported with the safety/efficacy outcomes. The most frequently reported combinations are in green, while the least reported are in red. On the other hand, Panel B displays the reporting frequencies of the PK metrics and outcomes while Panels C and D show the reporting patterns. For example, for T‐DM1, Cmax was reported together with DLT 24 times (red inset), consistent with the value in Panel A. Panel E indicates which cancers were investigated while Panel F shows the mg/kg doses for which each PK metric/outcome was reported. Data were extracted from 67 (T‐DM1) and 33 (T‐DXd) records, with studies using the same or overlapping datasets contributing data only once. The unit of analysis (T‐DM1: 55; T‐DXd: 57) was defined based on cancer type and dosing arm, with each cancer‐dose combination analyzed individually. When multiple cancers were investigated but not reported separately, they were analyzed together. The PK metrics and outcomes apply to the antibody–drug conjugates (T‐DM1 or T‐DXd). Abbreviations: AUC, area under the time‐concentration curve (cycle 1); AUC_inf, AUC extrapolated to infinity; Cmax, maximum concentration; Cmin, minimum concentration; DLT, dose‐limiting toxicity; ER, exposure response; ORR, objective response rate; OS, overall survival; PFS, progression‐free survival; PK, pharmacokinetic; T‐DM1, Trastuzumab emtansine; T‐DXd, Trastuzumab deruxtecan.

To identify clinical trials for our analysis, we conducted a search in MEDLINE and other relevant resources, as illustrated in Figure S1. A total of 651 studies underwent screening based on their titles and abstracts, and eventually, 103 studies were selected for inclusion. Of these, 72 studies (70%) were used for T‐DM1 analysis, including 14 studies for Pop‐PK modeling (details in Table S1 and Figure S2) and 67 studies for exposure–response (ER) analysis (Table S2 and Figure 2). For T‐DXd analysis, 34 studies (33%) were included, with four studies used for Pop‐PK analysis (Table S3 and Figure S2) and 33 studies for ER analysis (Table S4 and Figure 2). Three studies (2.9%) reported data on both T‐DM1 and T‐DXd. As shown in Figure 2, breast cancer was the most frequently reported cancer for T‐DM1 (89%, 49 of 55 units of analysis, where a unit of analysis is a cancer‐dose combination). For T‐DXd, the most reported cancers were breast cancer (33%, 19 of 57), colorectal cancer (11%, 6 of 57), non–small‐cell lung cancer (11%, 6 of 57), and gastric/gastroesophageal junction cancer (9%, 5 of 57). The percentage reporting of PK metrics and outcomes is shown in Panel B of Figure 2 while Figure S3 highlights the proportion of missing PK metrics and outcomes across the different dose levels.

### 3.2 Population Pharmacokinetic Models

Table 1 shows the parameter estimates of the fitted two‐compartment ADC models, which were consistent with previous reports. Nonlinear clearance and payload models were also tested, but we could not fit the parameters with acceptable precision (defined as relative standard errors [RSEs] of < 30% for fixed effect parameter estimates and < 50% for random effect parameter estimates). In the sensitivity analysis, only the DM1 model converged when BTAV was modeled separately from BSV (Table S5), with parameter estimates similar to the primary analysis. However, not treating study arms as separate studies reduced the effective sample size, causing some RSEs to exceed 300%, supporting our primary approach of modeling BTAV within BSV. Restricting the analysis to dose‐finding studies (Phase 1 trials reporting at least two dose levels) also produced similar parameter estimates but with reduced precision (Table S6).

**TABLE 1:** Pharmacokinetic parameter estimates of the structural models.

Parameter (units)	Mean estimate (RSE)	
	Trastuzumab‐emtansine	Trastuzumab‐deruxtecan
Clearance (L/day)	0.809 (7.3%)	0.585 (3.7%)
Central volume of distribution (L)	3.283 (4.8%)	2.785 (1.5%)
Peripheral volume of distribution (L)	0.748 (8.3%)	1.243 (9.2%)
Intercompartment clearance (L/day)	1.120 (26.3%)	0.652 (9.7%)
BSV clearance	0.334 (15.5%)	0.084 (48.3%)
BSV central volume of distribution	0.221 (14.8%)	0.038 (38.8%)
BSV clearance ~ BSV central volume of distribution	0.825 (10.1%)	0.915 (36.8%)
Weighted^a^ proportional error (%)	0.430 (9.1%)	0.338 (13.4%)
Constant error (ng/mL)	2633.766 (6.1%)	3043.004 (8.2%)

Abbreviations: BSV, between‐study variability; L, liter; RSE, relative standard error.

^a^ Proportional error was weighted by the square root of the sample size.

Except for a few outliers (e.g., lower concentrations around 400 h), prediction‐corrected visual predictive checks (Figure S4) indicated acceptable model fits, particularly for concentrations near Cmax. Consistent with these results, model simulations using Bayesian individual dynamic predictions (1000 replicates per dose; median weights: 69.4 kg for T‐DM1 and 59.0 kg for T‐DXd) accurately predicted exposures, particularly for Cmax (Figure S5). Given its accuracy, Cmax was selected for the clinical utility index (CUI) analysis, with Cmin and AUC also explored.

### 3.3 Exposure–Response (ER) Analysis

As shown in Figure 2, 11 studies provided both AUC cycle 1 (or AUC) and AUC_inf for T‐DM1, with AUC ratio (AUC/AUC_inf) median of 0.98 (interquartile range 0.97–0.99). This ratio was used to estimate AUC for 11 additional studies that reported AUC_inf but not AUC. For T‐DXd, all studies reporting AUC_inf also reported AUC, eliminating the need for imputation. After imputing other missing PK exposures using median values from other dose levels, ER analyses (Figure S6) identified the objective response rate (ORR) and dose‐limiting toxicity (DLT) as outcomes for progressing to clinical utility analysis (CUI) for both T‐DM1 and T‐DXd. Limited data availability (insufficient range for ER analysis) excluded overall survival (T‐DM1 alone) and progression‐free survival (both T‐DM1 and T‐DXd) from the analysis (Figure 2, Panel F). Overall survival for T‐DXd was reported for most doses; however, it showed a negative correlation with PK exposure (Figure S6, Panel B). This is likely due to shorter follow‐up in earlier trials—later trials that used higher doses had more deaths due to longer follow‐up.

### 3.4 Clinical Utility Index (CUI)

**FIGURE 3:**
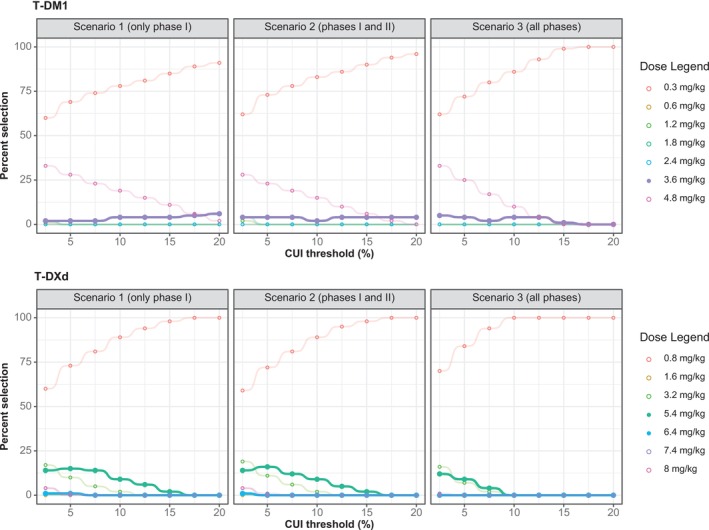
Impact of CUI thresholds on dose selection for T‐DM1 and T‐DXd. This figure shows the effect of varying CUI thresholds (2.5%–20%) on dose selection for T‐DM1 (Panel A) and T‐DXd (Panel B) in three scenarios. Increasing the CUI threshold, defined as the minimum percentage improvement in average CUI needed to select a higher dose, resulted in the selection of starting doses (0.3 mg/kg for T‐DM1 and 0.8 mg/kg for T‐DXd). The approved doses of 3.6 mg/kg (T‐DM1) and 5.4 and 6.4 mg/kg (T‐DXd) are depicted using solid circles. At each CUI threshold, weights ranging from 10% to 90% were tested. Abbreviations: CUI, clinical utility index; T‐DM1, trastuzumab emtansine; T‐DXd = trastuzumab deruxtecan.

For the CUI analyses, we selected Cmax (PK metric), ORR (efficacy), and DLT (safety) based on simulation accuracy (Figure S5) and the data range (Figure S6). We tested various ORR weights (10% to 90%, with DLT weight as 100% minus ORR weight) and CUI thresholds (2.5% to 20%). As shown in Figure 3, in all scenarios, increasing the CUI threshold (the minimum percentage improvement in average CUI needed to select a higher dose) increased the selection frequency of starting doses (0.3 mg/kg for T‐DM1 and 0.8 mg/kg for T‐DXd). Detecting significant CUI improvements or desired effect sizes (e.g., 20% for T‐DM1 and 10% for T‐DXd) became more challenging when later trial phases (III and IV) were included, compared to analyses of only Phase I (87% of which were dose finding for T‐DM1 and 80% for T‐DXd) and Phase II trials. CUI analyses using AUC (both ADCs) and Cmin (T‐DXd only) are shown in Figure S7 (T‐DM1 with Cmin as the PK metric is not shown due to insufficient data; Cmin was reported for only 3.6 mg/kg Figure 2).

**FIGURE 4:**
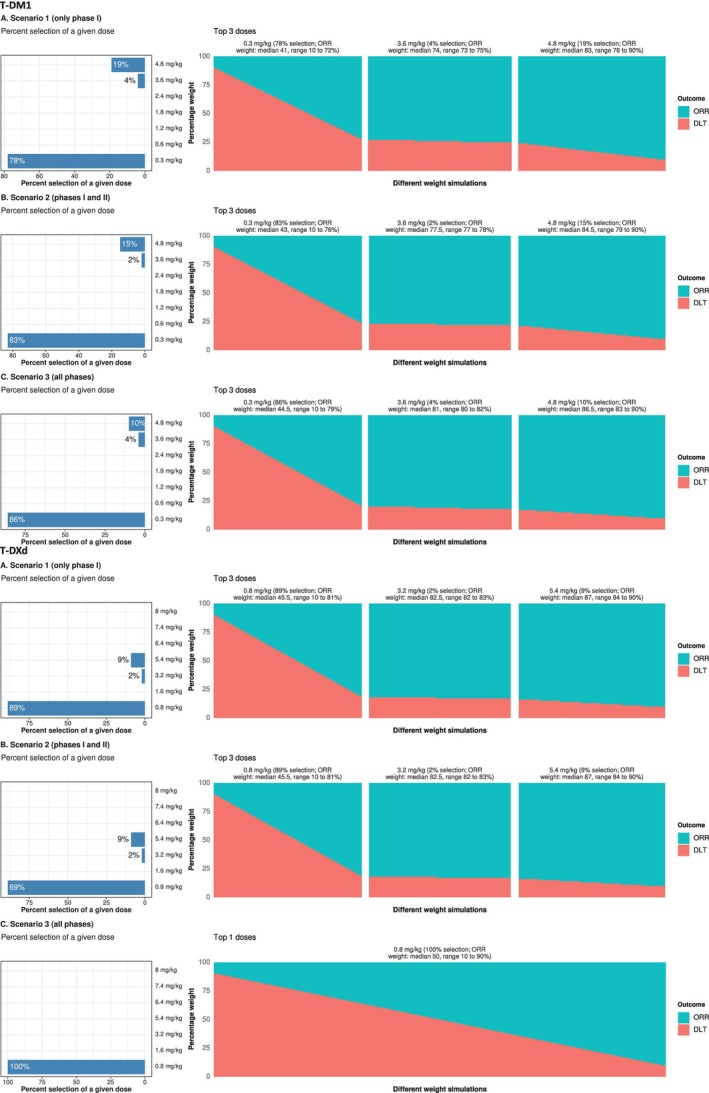
Weighting impact on dose selection for T‐DM1 and T‐DXd with a CUI threshold of 10%. This figure illustrates the effect of varying ORR and DLT weights (10% to 90%) on the selection of T‐DM1 and T‐DXd doses in Scenario 1 (only Phase I trials, Panel A), Scenario 2 (Phase I and II trials, Panel B), and Scenario 3 (all trial phases, Panel C), using a CUI threshold of 10% (at least a 10% improvement in average CUI needed to select a higher dose). The left panels show the frequency of selection for different dose levels, while the right panels provide details of the top (most) selected doses. Abbreviations: CUI, clinical utility index; DLT, dose‐limiting toxicity; ER, exposure response; ORR, objective response rate; T‐DM1, trastuzumab emtansine; T‐DXd, trastuzumab deruxtecan.

Detailed analyses (snapshots of specific CUI thresholds from Figure 3) were conducted at an arbitrary CUI threshold of 10%, and the results are shown in Figure 4. The approved 3.6 mg/kg T‐DM1 dose was selected in 4% of the different weight simulations when all trial phases were considered, with a median ORR weight of 81%. For T‐DXd, none of the two approved doses (5.4 and 6.4 mg/kg) were selected at a CUI threshold of 10% when all trial phases were considered. However, when only Phase I trials or only Phase I and II trials were considered, the 5.4 mg/kg dose was selected in 9% of the scenarios. Lower CUI thresholds would also select this dose, even when all trials are included, as shown in Figure 3.

**FIGURE 5:**
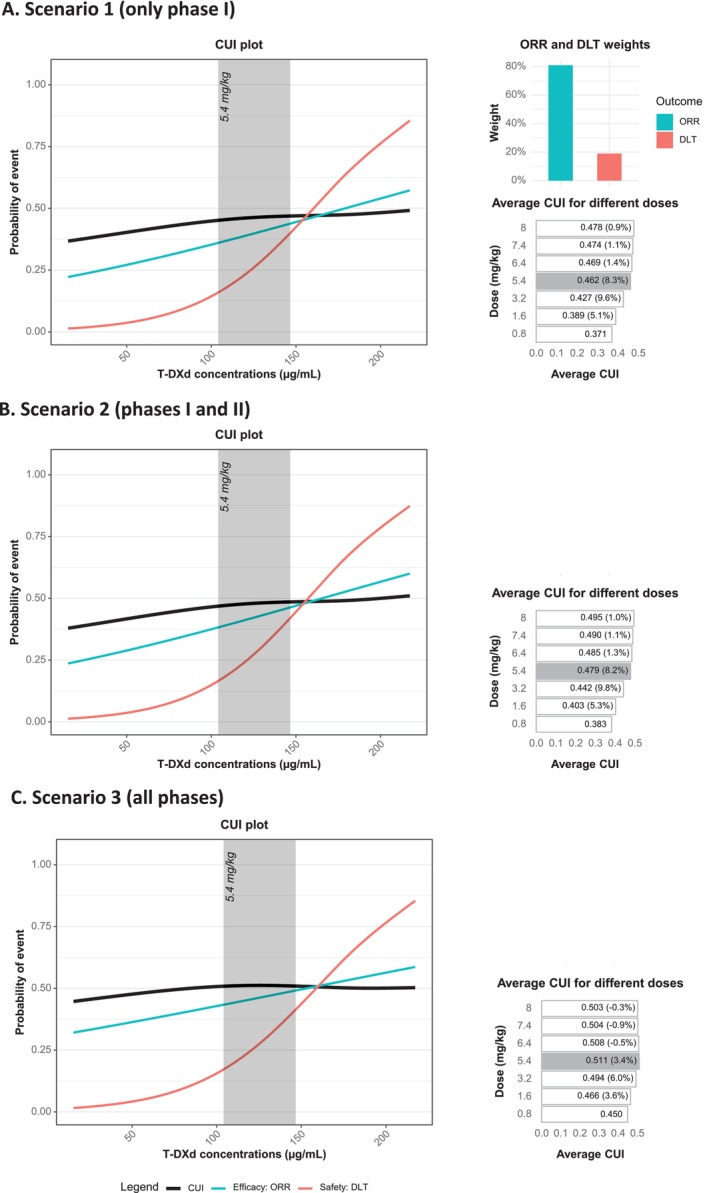
Dose selection for T‐DXd using R Shiny and a priori weights derived from T‐DM1. R Shiny and weights of 81% (ORR) and 19% (DLT), which were required to select the approved T‐DM1 dose (3.6 mg/kg) were used for T‐DXd. The figure shows the resulting selection of the approved 5.4 mg/kg dose of T‐DXd for breast cancer. The improvement in CUI compared to the 3.2 mg/kg dose is 8.3% in Scenario 1 (Panel A), 8.2% in Scenario 2 (Panel B), and 3.4% in Scenario 3 (Panel C). The regions corresponding to the selected doses are shaded gray. A CUI threshold (desired minimum effect size) of 2.5% was assumed for all scenarios. The annotations in the “Average CUI for different doses” plots represent the average CUI, calculated as CUI AUC/exposure range, with the percentage improvement (all dose levels except the starting dose) compared to the previous dose level shown in parentheses. Although not shown here, other PK metrics (AUC and Cmin), PFS, and principal component analysis weighting are included in the Shiny app for exploration purposes. For visual clarity, the labels use μg/mL instead of the ng/mL used during analysis. Abbreviations: AUC, area under the time–concentration curve (Cycle 1); Cmax, maximum concentration; Cmin, minimum concentration; CUI, clinical utility index; DLT, dose‐limiting toxicity; ER, exposure response; ORR, objective response rate; PFS, progression‐free survival; PK, pharmacokinetic; T‐DM1, trastuzumab emtansine; T‐DXd, trastuzumab deruxtecan.

In real‐world applications, weights must be determined in advance, which can be guided by information from previously approved drugs (e.g., T‐DM1, approved earlier, could guide dose selection for T‐DXd). To emulate this process, we used the T‐DM1 weight of 81% for ORR (and the corresponding DLT weight of 19%). Applying these weights effectively selected the approved T‐DXd dose for breast cancer and non–small‐cell lung cancer (5.4 mg/kg, Figure 5). The improvement in CUI compared to the 3.2 mg/kg dose was 8.3%, 8.2%, and 3.4% in Scenario 1 (only Phase I trials), Scenario 2 (Phase I and II trials), and Scenario 3 (all trial phases), respectively.

## 4 Discussion

We conducted a systematic review of the literature to select clinical studies and develop Pop‐PK models for two trastuzumab drug conjugates: T‐DM1 and T‐DXd. The key parameter estimates of the fitted two‐compartment models were similar to those previously estimated by Lu et al. [20] for T‐DM1 and Yin et al. [19] for T‐DXd. Our results focus on the conjugated ADCs as limited data prevented precise modeling of the payloads. Nevertheless, the International Consortium for Innovation and Quality in Pharmaceutical Development has reported that the conjugated ADC correlates well with efficacy and safety [26].

Summary‐level data from our systematic review supported exposure–response (ER) modeling for both drugs, with Cmax selected for clinical utility index (CUI) analysis. Previous studies [18, 21, 27, 28, 29] have typically used Cmin and AUC as pharmacokinetic (PK) metrics in ER analyses. However, we prioritized Cmax due to its higher simulation accuracy and wider reporting (43.6% for T‐DM1 and 19.3% for T‐DXd; Figure 2). We reported (Figure S7) the results using Cmin and AUC, which should be interpreted cautiously. Regarding efficacy outcomes, we used the objective response rate (ORR), which was consistent with most previous analyses [18, 21, 27, 28, 29]. Unlike these previous studies, which analyzed safety outcomes such as serious adverse events, grade ≥ 3 adverse events, thrombocytopenia, and interstitial lung disease (ILD), we used a composite endpoint: dose‐limiting toxicity (DLT), encompassing DLT (if reported by the primary study), dose reduction, drug discontinuation, or drug interruption/delay. As explained in the methods section (Text S2), DLT is robust to interventions like rescue medications that might obscure the relationship between PK exposure and toxicity. It comprehensively captures adverse effects, including serious or grade ≥ 3 treatment‐related adverse effects or drug‐specific toxicities. For instance, we did not specifically analyze T‐DXd‐related ILD. However, ILD management protocols typically involve dose interruption (Grade 1 ILD) or discontinuation (Grades 2 and above) [14, 30] ensuring that patients experiencing ILD were included in our safety endpoint analysis.

To determine the optimal drug exposures and doses that maximize the benefit–risk balance, we combined model‐predicted ORR and DLT into a composite score, the CUI. We tested various CUI thresholds (2.5%–20%), finding that higher thresholds (close to 20%) led to the selection of the starting doses (0.3 mg/kg for T‐DM1 and 0.8 mg/kg for T‐DXd) with higher frequency. Dose selection also depended on the weights applied (10%–90%). While a wide range helps when the efficacy–safety balance is uncertain, it can lower the selection frequency of approved doses (e.g., emphasizing safety with high DLT weights consistently led to the selection of the lowest dose). Narrower ranges (e.g., 10%–30% for DLT or 70%–90% for ORR) may improve precision but require prior knowledge to avoid missing or misidentifying the optimal dose. In postapproval scenarios (using all available data) and with a CUI threshold of 10%, the approved dose was selected when ORR was given a median weight of 81% (approximately four times the weight of DLT). Notably, this weighting was lower in Scenario 1 (only Phase I trials, 74%) and Scenario 2 (Phase I and II trials, 77.5%), indicating that the desired balance of efficacy and safety for T‐DM1 shifted toward more emphasis on efficacy as the drug progressed through the development pipeline.

In practical settings, establishing weights in advance is essential, and this process can be guided by clinicians and domain experts, with insights from previously approved therapies (e.g., T‐DM1 approved earlier for breast cancer). Using the efficacy and safety balance from T‐DM1 for T‐DXd effectively identified the 5.4 mg/kg dose of T‐DXd for breast cancer. This dose showed an improvement in CUI compared to the 3.2 mg/kg dose by 8.3% in Scenario 1 (only Phase I trials), 8.2% in Scenario 2 (Phase I and II trials), and 3.4% in Scenario 3 (all trial phases). Of note is that all these improvements are below 10%. Therefore, using a 10% threshold would mean the 5.4 mg/kg dose would not be selected. This suggests that in clinical practice, thresholds should not be rigidly set; the choice of thresholds will depend on many factors, including the cancer subtype. This makes the involvement of clinicians and patients or their representatives crucial during decision‐making. Additionally, consistent with the results in Figure 3, which showed that including later trial phases makes it harder to detect large CUI increments, the CUI improvement was lowest for Scenario 3 (3.4%). This may reflect the inclusion of real‐world patients with greater heterogeneity, which can dilute the treatment effect. As illustrated in Figure S6, the ORR slope becomes less steep as one moves from Scenario 1 to Scenario 3, highlighting the impact of later‐phase trials on the overall efficacy measurement.

The 6.4 mg/kg dose, approved for gastric cancer, [14] was not selected at the 10% CUI threshold. Selecting this dose at lower thresholds would require shifting the balance further toward efficacy, implying a willingness to accept more toxicity to achieve higher efficacy. For example, Shitara et al. tested both 5.4 and 6.4 mg/kg doses in a T‐DXd dose expansion Phase 1 study for gastric cancer [31]. The results indicated that while the 5.4 mg/kg dose was safer (21% [4/19] vs. 28% [7/25] drug‐related treatment‐emergent adverse events leading to drug interruption), it was less efficacious (32% [6/19] vs. 52% [13/25] confirmed ORR). Supplemented by later trials, the higher dose was approved, highlighting the trade‐off between efficacy and safety in dose selection.

Although the CUI can accommodate multiple attributes, including patient‐reported outcomes [8, 11] we included only the ORR and DLT. Initially, we intended to use multiple efficacy outcomes, but other outcomes like progression‐free survival (PFS) and overall survival (OS) had insufficient data range (like the PK metric Cmin) and did not correlate with PK exposure. Unlike ORR [32] PFS and OS are longer term outcomes typically investigated in later‐phase studies, such as Phase III. When explored in earlier phases (I and II), shorter follow‐up times mean they may not be comparable with the longer follow‐up in Phase III. To allow exploration of an additional outcome, we have included PFS in a Shiny App (code available at https://github.com/iasiimwe/adc_cui). Additionally, we have included other PK metrics (AUC and Cmin) and an option to calculate weights using principal component analysis. Another limitation of this study is using only MEDLINE; however, MEDLINE has high coverage, estimated at 92.3% when compared with Google Scholar and EMBASE [33]. We also checked other resources, including ClinicalTrials.gov, Citeline, and the Beacon ADC database, to identify additional records. Moreover, this study aimed to demonstrate proof of concept and the applicability of MBMA and CUI in risk–benefit analysis; because postapproval evaluation is ongoing, any newly identified information can always be added. During simulations, we used median values (e.g., median weight), so the results are applicable to a typical subject. This approach is well accepted in pharmacometrics. Also, since we have shared the code used, simulations can be adjusted for atypical subjects by modifying variables such as weight. MBMA can integrate internal individual‐level data and summary‐level data from published clinical trials [7] and this additional information can help overcome some limitations of using study‐level data. These limitations include an insufficient sample size, which limited our ability to fit nonlinear models, develop models for the payload, and stratify by cancer types; variability in outcome definitions, such as differences in DLT criteria between early and late clinical development; different follow‐up times; and insufficient information on covariates, which limited our ability to conduct covariate testing.

In conclusion, we have shown that using trastuzumab–drug conjugates with the help of MBMA, a CUI approach can be valuable for postmarketing evaluations of ADCs. When we focused on pre‐Phase III trials, we showed how this approach can be used to select the doses that maximize the efficacy–safety balance during early drug development, and this could complement the Project Optimus principles [6]. Specifically, we used the results of T‐DM1's assessment to successfully predict the dose approved for T‐DXd for breast cancer. We have shared the code for a Shiny app that can be used to explore the effects of changing the components and weights included in the CUI that can be easily implementable for ADC drug developers or to guide clinical decisions. As reported, the CUI thresholds, or the minimum percentage improvement in average CUI needed to select a higher dose, also determine the final dose to be selected. The many key decisions—such as which PK, efficacy, and safety components to use, the weights to assign, the efficacy–safety balance to accept, and the CUI thresholds/desired effect size—highlight the importance of multidisciplinary collaboration, including input from clinicians and patients/patient representatives, during drug development. They also highlight the necessity of a systematic approach to quantitative assessment, such as the one presented in this paper. This proof‐of‐concept quantitative assessment of ADCs can result in time and cost savings for drug developers and optimize dosing to maximize patient benefit in clinical practice. While demonstrated with ADCs, this approach is broadly applicable to other drug modalities, offering a robust cost‐effective method for improving dose selection.

## Author Contributions

All authors wrote the manuscript and designed the research. I.G.A. and N.C. performed the research and analyzed the data. All authors contributed new reagents/analytical tools.

## Conflicts of Interest

Eunice Yuen, Raimund Peter, and Venkatesh Pilla Reddy are full‐time employees of and hold shares in Eli Lilly. Innocent Asiimwe and Nour Chtiba were Fellows as part of the APT fellowship collaboration project with Eli Lilly. All other authors declared no competing interests in this work.

## Supporting information




Data S1.

Data S2.

## Data Availability

All data generated or analyzed during this study are included in this published article and its Supporting Information files. All codes used during analysis are available at https://github.com/iasiimwe/adc_cui.
