# Supplementary material for: Postmarketing Assessment of Antibody–Drug Conjugates: Proof‐of‐Concept Using Model‐Based Meta‐Analysis and a Clinical Utility Index Approach
Source: CPT Pharmacometrics Syst Pharmacol. 2025 Mar 4;14(11):1810–22. doi: 10.1002/psp4.70013 (PMC12625083; doi:10.1002/psp4.70013)
Supplement: Supplementary file 1 — Data S1. [file PSP4-14-1810-s001.docx]

**Post-marketing Assessment of Antibody-Drug Conjugates: Proof-of-concept using Model-Based Meta-Analysis and a Clinical Utility Index Approach**

**Table of contents**

**Supplementary Text…..…………………………………………………………………………………………………….2**

**Supplementary Figures…………………………………………………………………………………………………….5**

**Supplementary References..………………………………………………………………………………………..…..15**

**Supplementary Text**

**Text S1: Systematic Review of the Literature to identify Clinical Trials**

To identify clinical trials for our analysis, we conducted a systematic search of MEDLINE through Ovid (<https://ospguides.ovid.com/OSPguides/medline.htm>) on 1^st^ May 2024, using the following search terms, limited to human trials: *(trastuzumab and (emtansine or t-dm1 or kadcyla or shr-a1201) or (deruxtecan or t-dxd or enhertu or ds-8201a) and (pharmacokinetic* or pharmacodynamic* or safety or efficacy)).mp*, where mp= “*title, book title, abstract, original title, name of substance word, subject heading word, floating sub-heading word, keyword heading word, organism supplementary concept word, protocol supplementary concept word, rare disease supplementary concept word, unique identifier, synonyms, population supplementary concept word, anatomy supplementary concept word*”. To identify more eligible trials, we searched through the reference lists of the studies we found, as well as previous systematic reviews. We also looked through resources such as ClinicalTrials.gov, clinical pharmacology/biopharmaceutics reviews, approved labels, sponsor application summary documents, the antibody-drug conjugates database^1^, Citeline (<https://www.citeline.com/en>), and the Beacon ADC database (<https://beacon-intelligence.com/solutions/adc/>).

The study selection criteria were:

a) Observational and interventional clinical studies that involved patients treated with either T-DM1 or T-DXd as monotherapy.

b) The studies had to provide dosing information.

c) The studies had to report at least one of the following: ADC/payload concentrations over time, efficacy data (with a priority given to confirmed responses in terms of objective response rate, overall survival, or progression-free survival), and safety data (including dose-limiting toxicity, dose reduction, drug discontinuation due to toxicity, or drug interruption/delay).

We did not limit studies based on indication, clinical phase, year of publication, or publication status. However, studies reported in non-English languages were excluded unless a translated version was available.

Two authors of this paper (IGA and NC) screened the titles, abstracts, and full texts of the retrieved bibliographic records for eligibility. The records for T-DM1 were reviewed once, while the records for T-DXd were independently reviewed by both reviewers. Any disagreements were resolved by consensus. A data extraction template was created to gather important information on study and patient characteristics, outcomes, and summary-level results. For PK results presented in the figures, we used an online tool (<https://automeris.io/WebPlotDigitizer.html>, version 4) to digitize and extract the data. We identified studies utilizing the same or overlapping datasets by examining study acronyms, clinical trial registration numbers, recruitment sites, authors, and their affiliations. Information from studies using the same/overlapping datasets was included only once to avoid duplicating subjects in the analysis. When selecting a study from a group of similar studies, we prioritized those that: a) stratified outcomes by PK exposure, b) reported outcomes by cancer type (if multiple cancers were investigated, each cancer type was analyzed separately), c) published primary results, and d) reported the highest number of subjects or the most detailed results. In studies that reported assessments by both independent review committees and investigators, we prioritized the results from the independent review committees.

**Text S2: Rationale for selecting dose-limiting toxicity (DLT) as the preferred safety endpoint and how DLT events were combined.**

We did not consider other safety endpoints, such as serious adverse events or grade ≥3 treatment-related adverse events, since previous analyses of T-DM1 and T-DXd have shown that these do not correlate well with PK exposure.^2-4^ As a molecule progresses from phase I to phase III, cumulative experience with the drug PK/PD and efficacy/safety data increases, making adverse events easier to identify and manage (e.g., using rescue medications, dose reductions, or treatment delays). This may prevent progression to grade 3 and above, potentially explaining the lack of correlation between PK exposure and serious adverse events. Since serious or grade ≥3 treatment-related adverse effects can lead to dose reduction, drug discontinuation, or drug interruption/delay, patients experiencing these events were likely captured in our analysis using the DLT endpoint described above.

Additionally, using a composite endpoint increased the number of events, addressing the challenge of fitting logistic regression models for individual endpoints including serious adverse events or grade ≥3 treatment-related adverse events, which are less frequently reported. Nevertheless, all safety endpoints can be analysed, particularly when rich individual-level datasets are available (e.g., internally).

To combine DLT events and select the most frequent occurrence of any of the following: DLT (if reported by the primary study), dose reduction, drug discontinuation, or drug interruption/delay, we used a function (DLT_fn, see “*3.ER analysis.R*” at <https://github.com/iasiimwe/adc_cui>): *function (x, y) if(length(unique(x)) == 1 & TRUE %in% is.na(unique(x))) return(NA) else return(y[which.max(x)])*. Briefly, this function,

1. Checks for missing data: If all values in x are identical and one of them is NA (missing), the function returns NA. This handles rows where no relevant data is available for these outcomes.
2. Returns the "Worst-Case" outcome: if the condition above is not met, the function identifies the index of the maximum value in x using which.max(x) and returns the corresponding value from y.

To obtain the sample size (N) for the "Worst-Case" outcome, x is a vector that contains the number of events (for DLT, dose reduction, drug discontinuation, or drug interruption/delay), while y is a vector containing the corresponding sample sizes for these outcomes. To calculate the number of events (E) for the "Worst-Case" outcome, both x and y are vectors containing the number of events for DLT, dose reduction, drug discontinuation, or drug interruption/delay.

**Text S3: Key Steps in Developing a Clinical Utility Index (CUI)**

1. *Identify Key Components*
   Select the most relevant safety and efficacy outcomes. For example, we chose objective response rate (ORR) as a measure of efficacy and dose-limiting toxicity (DLT) as a measure of safety.
2. *Assign Weights to Each Component*
   Use expert opinions, stakeholder input, or statistical methods (e.g., principal component analysis) to determine the relative importance of each component. In this study, we tested weights ranging from 10% to 90%, avoiding 0% and 100% since no approved drugs exhibit purely beneficial or purely adverse effects. For the example below, equal weights (50% each) were assigned to the probability of ORR (pORR) and the probability of not experiencing DLT (1 – pDLT). This (1 – pDLT) ensures all utilities are aligned in a beneficial direction.
3. *Normalize Components to a Common Scale*
   Convert all components to a scale ranging from 0 to 1. Since both pORR and pDLT are already probabilities, this step was unnecessary in our case.
4. *Combine Components into a Composite Score*
   Compute the composite CUI score using the formula: ***U(x₁, ..., xₙ) = ∑wᵢUᵢ(xᵢ)*,** where xᵢ represents the attribute values, the weights (wᵢ) sum to 1, and each utility function (Uᵢ) is scaled between 0 and 1. Assuming equal weights (0.5 each), the CUI is calculated as: ***CUI = (pORR * 0.5) + ((1 – pDLT) * 0.5)***.
5. *Calculate Dose-Associated CUI Using PK Metrics*
   When applying a pharmacokinetic (PK) exposure-based approach, multiple PK exposures (e.g., AUC, Cmax, or Cmin) are associated with a single dose. This yields several CUI values across the exposure range for that dose. To determine a single CUI value for the dose, calculate the average CUI by integrating the CUI curve over the PK exposure range and dividing by the range. For example, if the AUC for dose A ranges from AUC₁ to AUCₙ, this formula provides the average CUI value for dose A: ***U_A_(AUC_1_, ..., AUC_n_) = (∑Uᵢ(AUCᵢ)) / ( AUC_n_- AUC_1_)***. For integration, we used the trapezoidal rule expressed as: ***sum(diff(x) * (y[-1] + y[-length(y)]) / 2)***, where x represents the PK exposure (e.g. AUC) values on the x-axis and y represents the corresponding CUI values on the y-axis.

**Supplementary Figures**

Included records (*n* = 103)

Unique MEDLINE-indexed records identified on 1^st^ May 2024 (*n* = 577)

Records screened by reviewing titles and/or abstracts (*n* = 651)

Full-text records assessed for eligibility (*n* = 237)

**Identification**

**Screening**

**Eligibility**

**Included**

Additional records:

- ClinicalTrials.gov (*n* = 61)
- Identified through lists of references (*n* = 13)

Full-text records excluded, with reasons (*n* = 134)

- No PK, dose and/or outcome information = 53
- ClinicalTrials.gov record with an already included published study = 31
- T-DM1/T-DXd combination therapy = 24
- Did not evaluate T-DM1/T-DXd = 11
- Case report(s) = 9
- Reviews, guidelines, comments, editorial, secondary studies such as cost effectiveness studies etc. = 4
- Preclinical studies including assay development = 2

Records excluded, with reasons (*n* = 414)

- Reviews, guidelines, comments, editorial, secondary studies such as cost effectiveness studies etc. = 228
- Preclinical studies including assay development = 129
- Did not evaluate T-DM1/T-DXd = 23
- T-DM1/T-DXd combination therapy = 19
- Protocol = 11
- Case report(s) = 3
- No PK or dose information = 1

T-DM1 (*n* = 72)^b^

- Pop-PK analysis = 14
- ER analysis = 67

T-DXd (*n* = 34)^b^

- Pop-PK analysis = 4
- ER analysis = 33

**Figure S1. Flow chart of included records.** ^a^The sum of included records (103)^2-104^ is less than 106 (72 + 34) since three records reported both T-DM1 and T-DXd data.  ^b^Nine (T-DM1) and three (T-DXd) records reported both Pop-PK and ER analysis data, respectively. Abbreviations: ER = exposure response, Pop-PK = population pharmacokinetic, T-DM1 = Trastuzumab emtansine, T-DXd = Trastuzumab deruxtecan.

**A.** **T-DM1 (antibody-drug conjugate)**

**
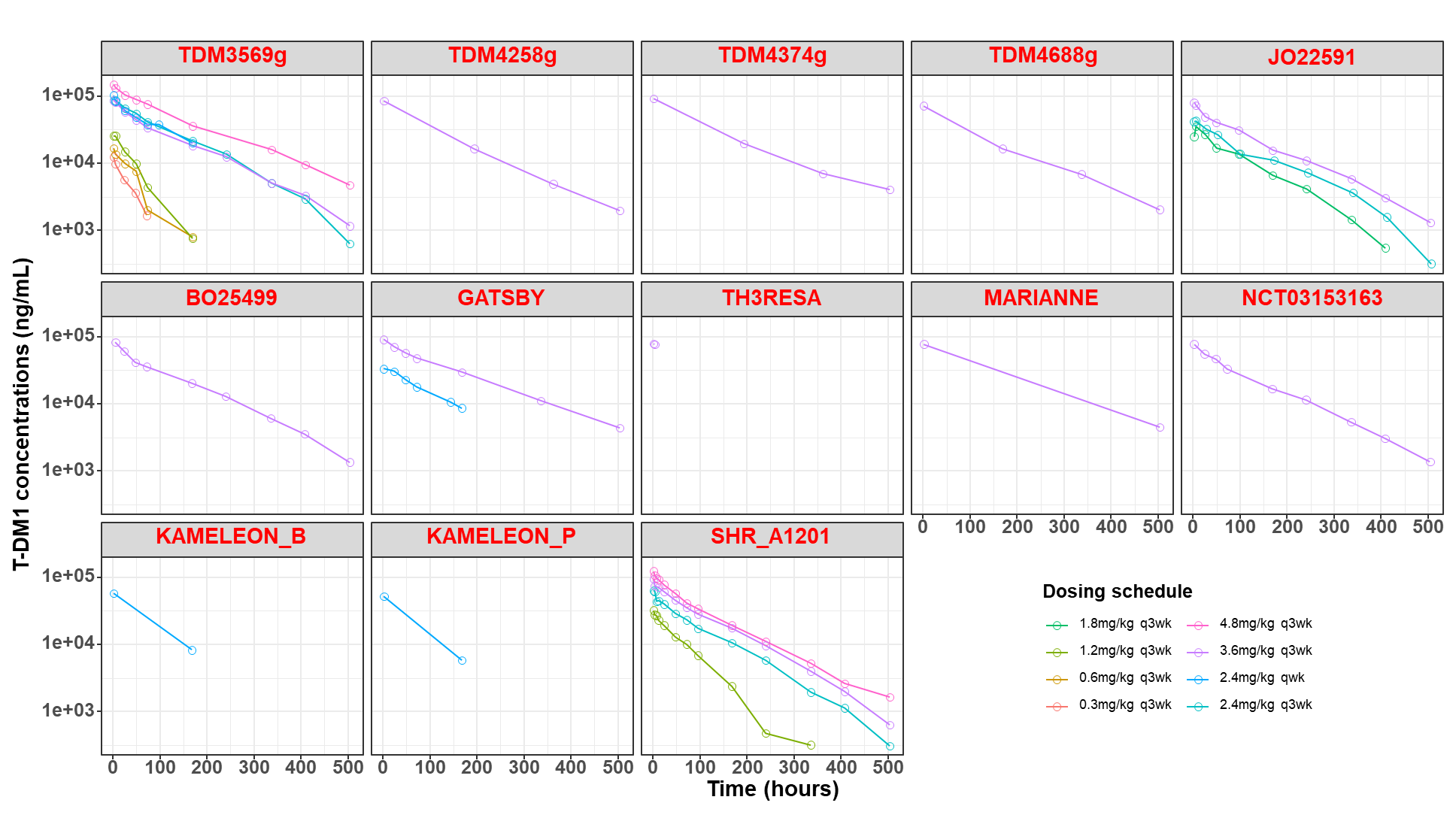
**

**B.** **DM1 (payload)**

**
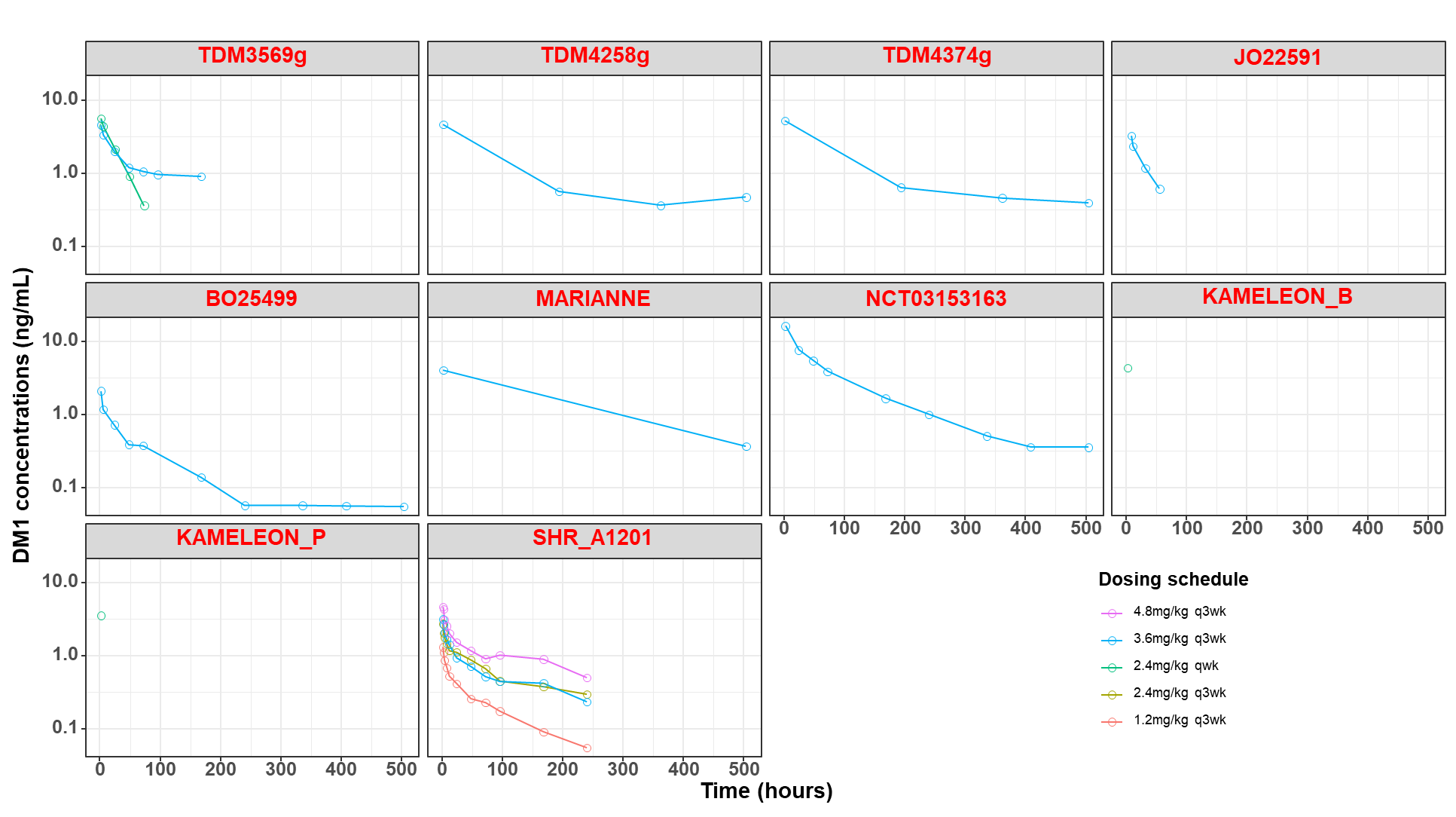
**

**C.** **T-DXd (antibody-drug conjugate)**

**
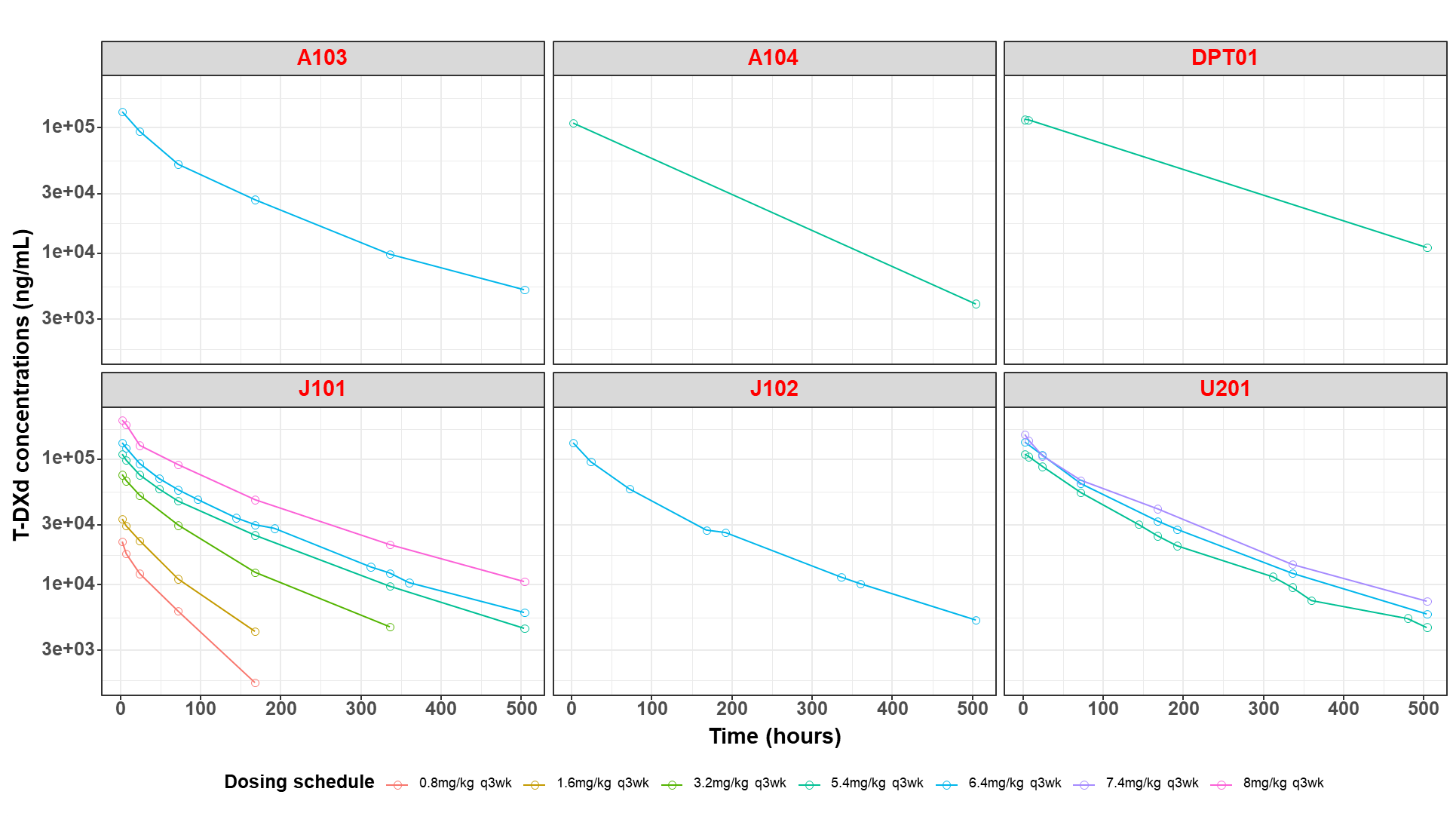
**

**D. DXd (payload)**

**
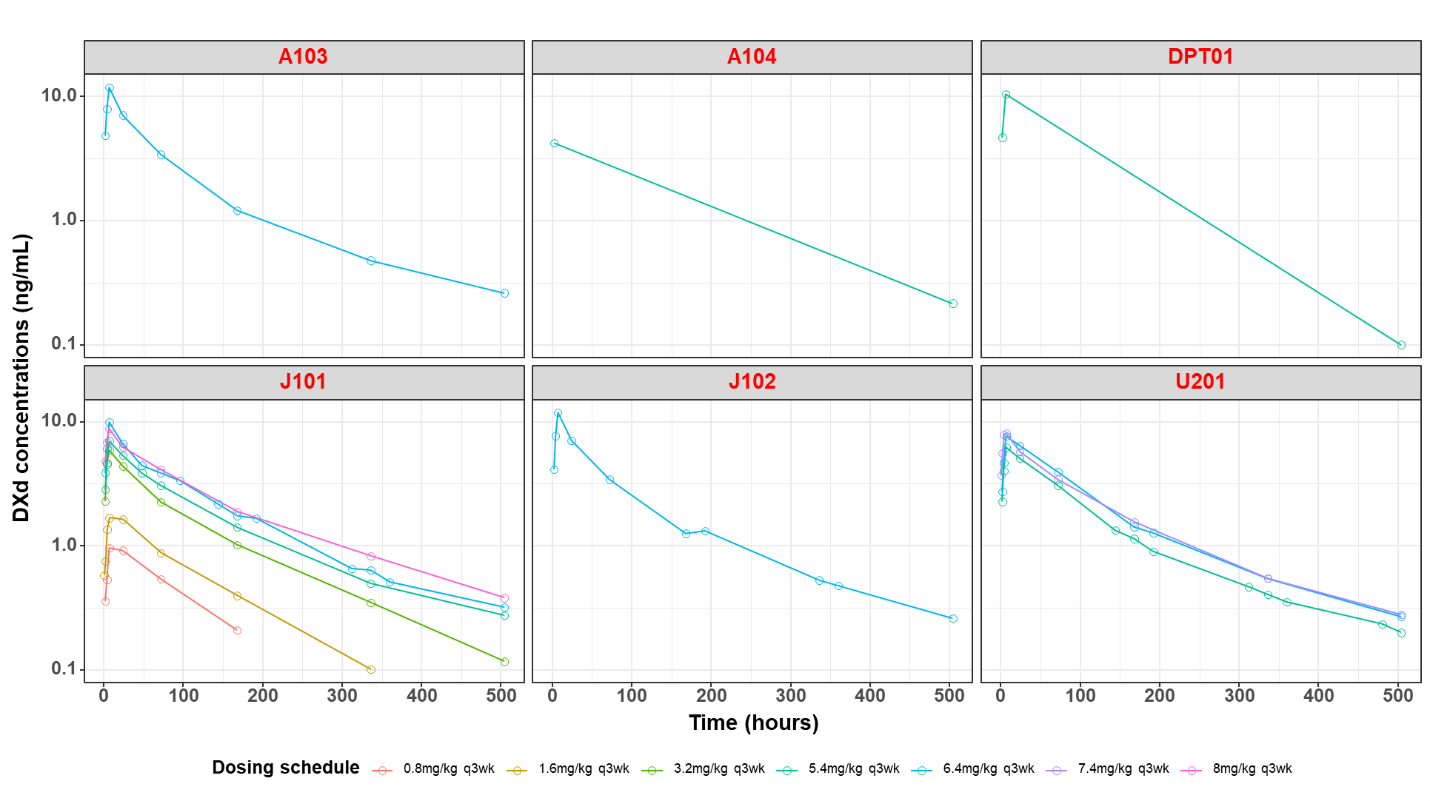
**

**Figure S2. Digitized concentration-time profiles of the Trastuzumab drug conjugates and their payloads. A.** T-DM1, **B.** DM1, **C.** T-DXd, **D.** DXd. Data was extracted from fourteen records^4-17^ for T-DM1 and DM1, and from four records^18-21^ for T-DXd and DXd. Plots were extracted/digitized using WebPlotDigitizer (<https://automeris.io/WebPlotDigitizer.html>, version 4). DMI = emtansine, DXd = deruxtecan, qwk = weekly dosing, q3wk = three-weekly dosing, T-DMI = Trastuzumab emtansine, T-DXd = Trastuzumab deruxtecan.

**A. T-DM1**

**
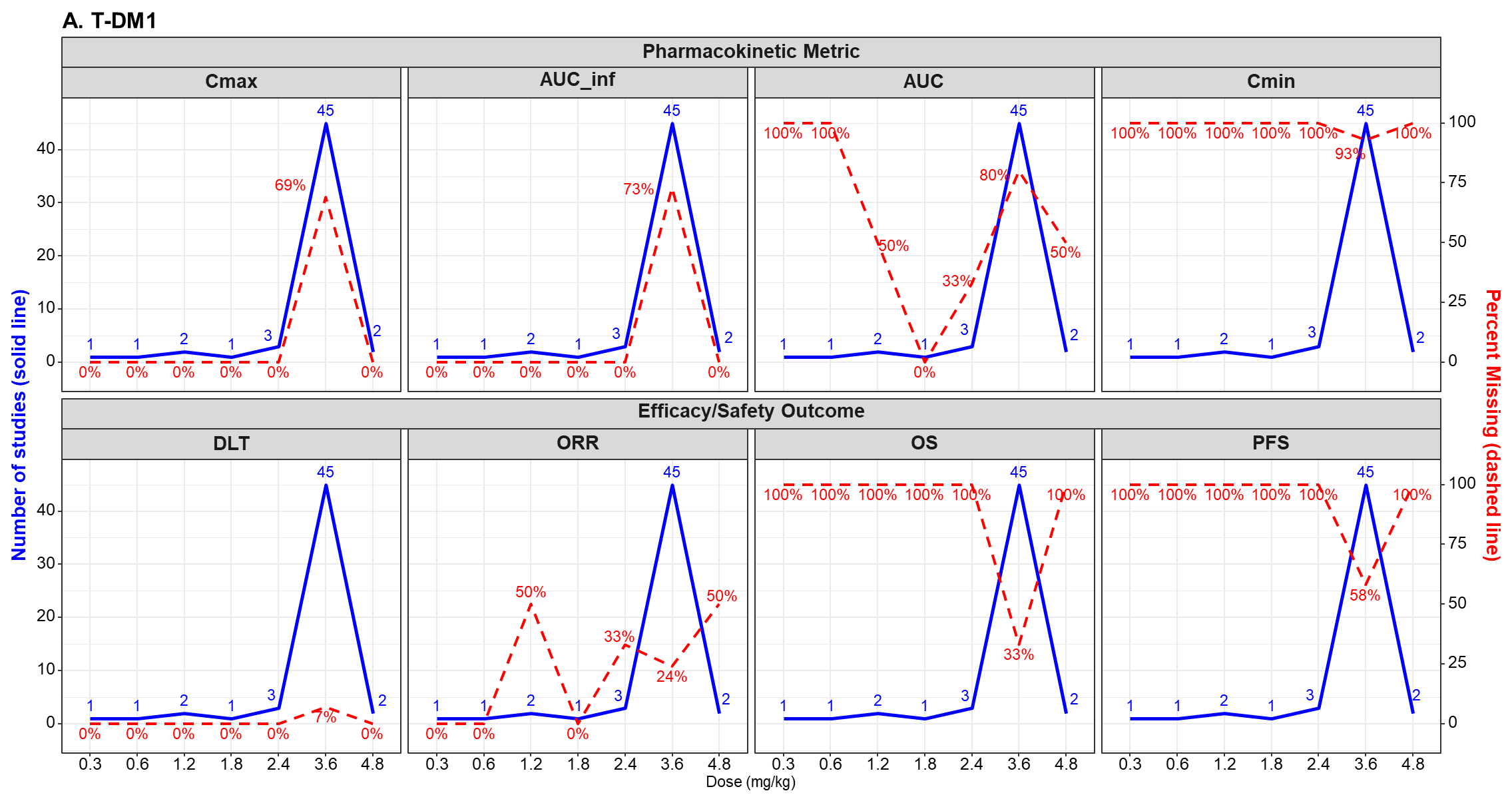
**

**B. T-DXd**


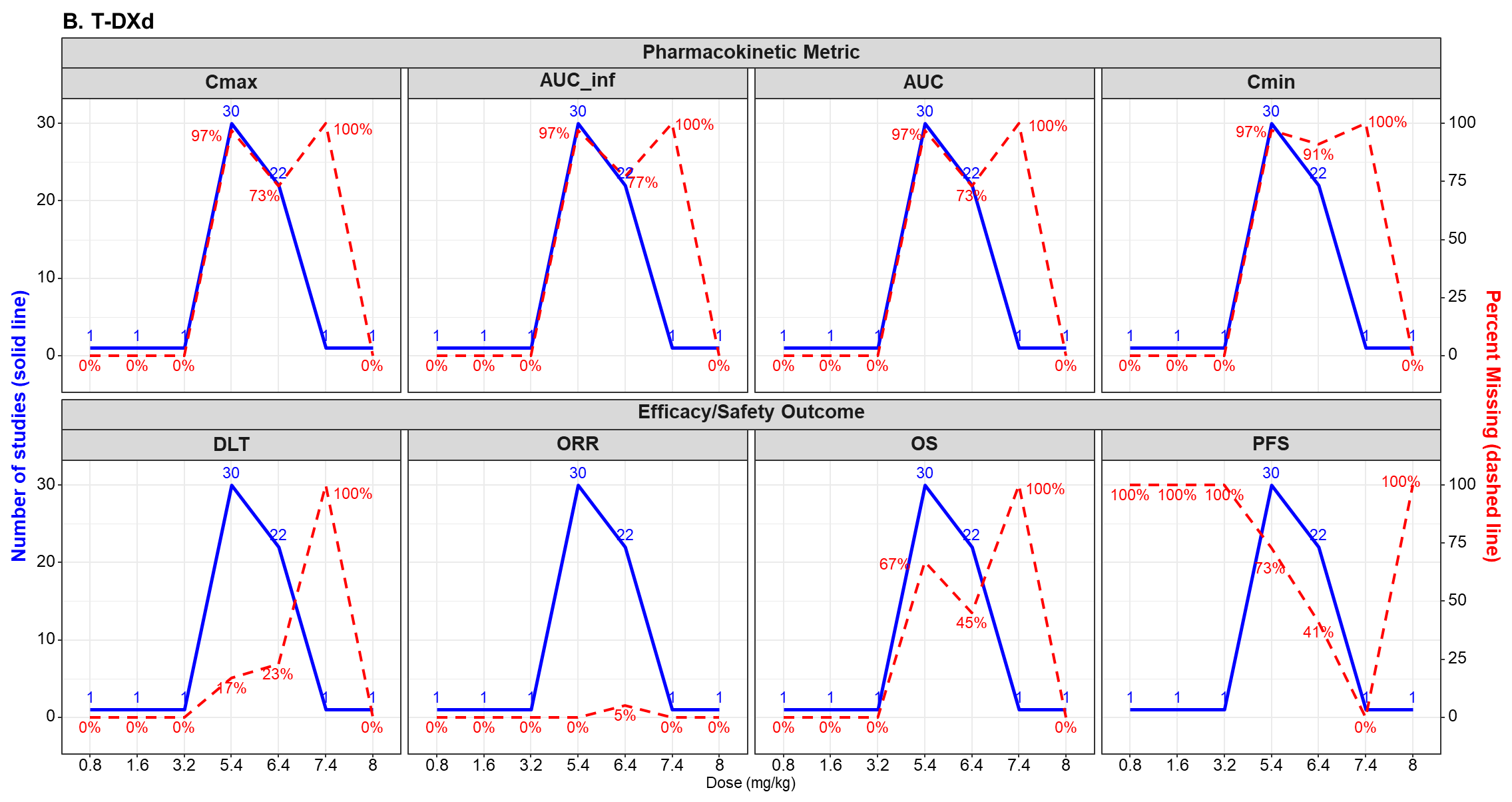


**Figure S3. Proportion of missing PK metrics and outcomes across dose levels. A.** T-DM1 **B.** T-DXd. The blue solid line shows the total number of analysis units at each dose level (55 for T-DM1 and 57 for T-DXd) while the red dashed line indicates the percentage of missing data for each dose level. For T-DM1, the most reported dose level was 3.6 mg/kg (45 analysis units). At this dose level, Cmax was the most frequently reported PK metric (missing in 69% of analysis units), and DLT the most frequently reported outcome (missing in only 7% of analysis units). An analysis unit was defined as a unique cancer type and dosing arm combination. When multiple cancer types were investigated but not reported separately, they were analysed together. AUC = area under the time-concentration curve (cycle 1), AUC_inf = AUC extrapolated to infinity, Cmax = maximum concentration, Cmin = minimum concentration, DLT = dose-limiting toxicity, ER = exposure response, ORR = objective response rate, OS = overall survival, PFS = progression-free survival, PK = pharmacokinetic, T-DMI = Trastuzumab emtansine, T-DXd = Trastuzumab deruxtecan.


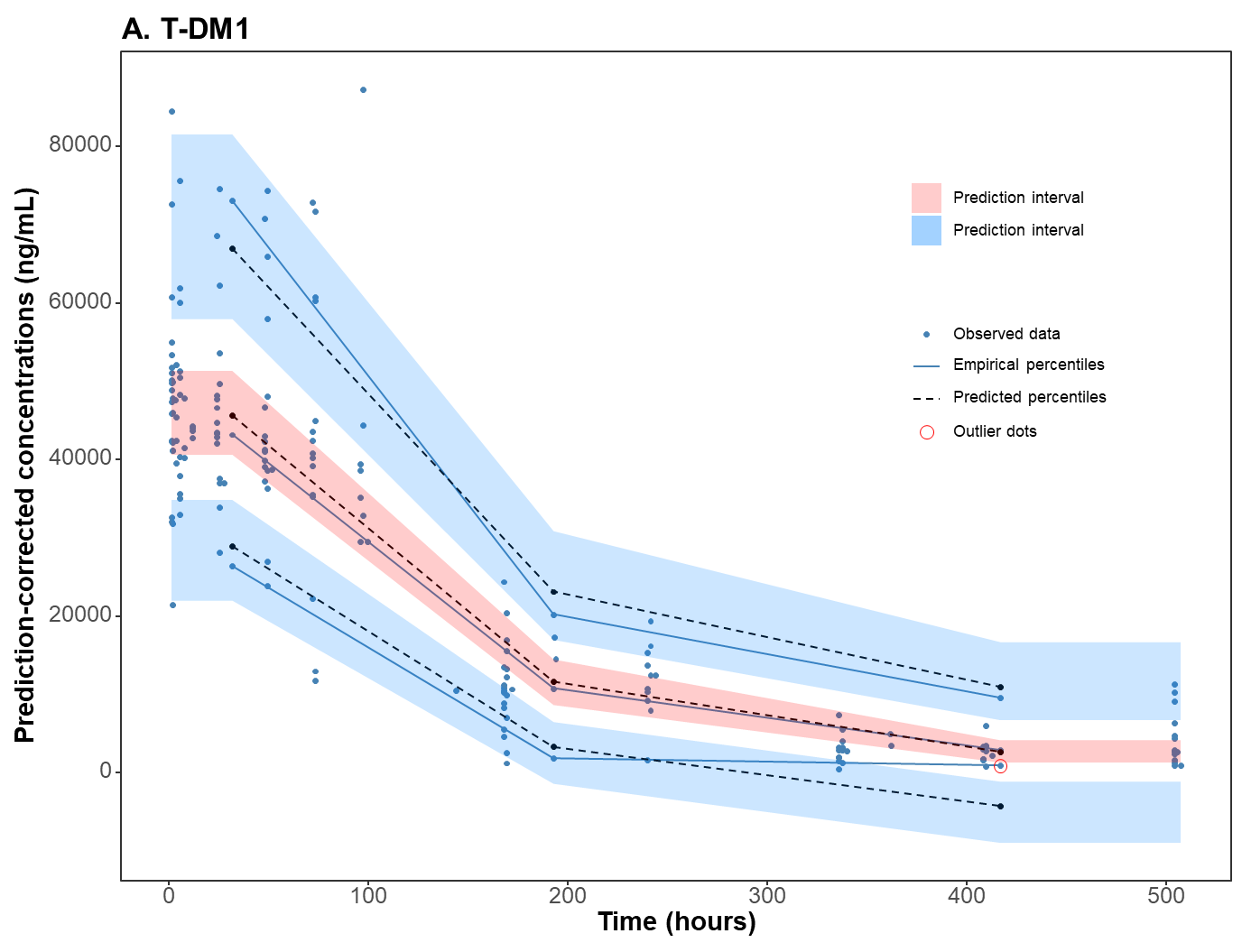


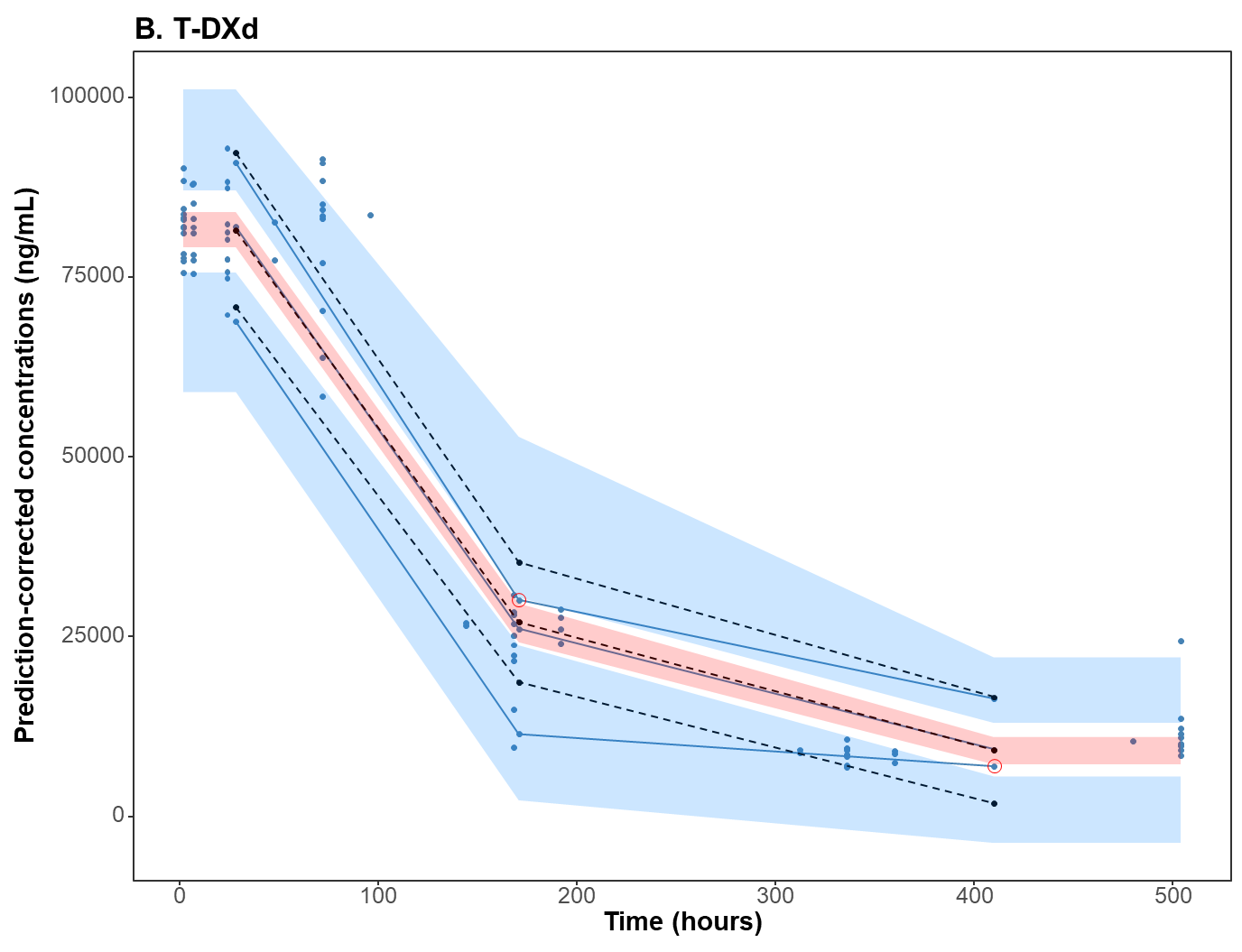


**Figure S4.** **Visual predictive checks of the concentration-time profiles of the Trastuzumab-drug conjugates. A.** Trastuzumab emtansine (T-DM1). **B.** Trastuzumab deruxtecan (T-DXd). The prediction intervals represent 95% confidence intervals. Empirical percentiles indicate the percentiles of the observed data, while predicted percentiles are based on the model’s simulations. Outlier dots represent individual observed data points that fall outside the predicted percentiles.


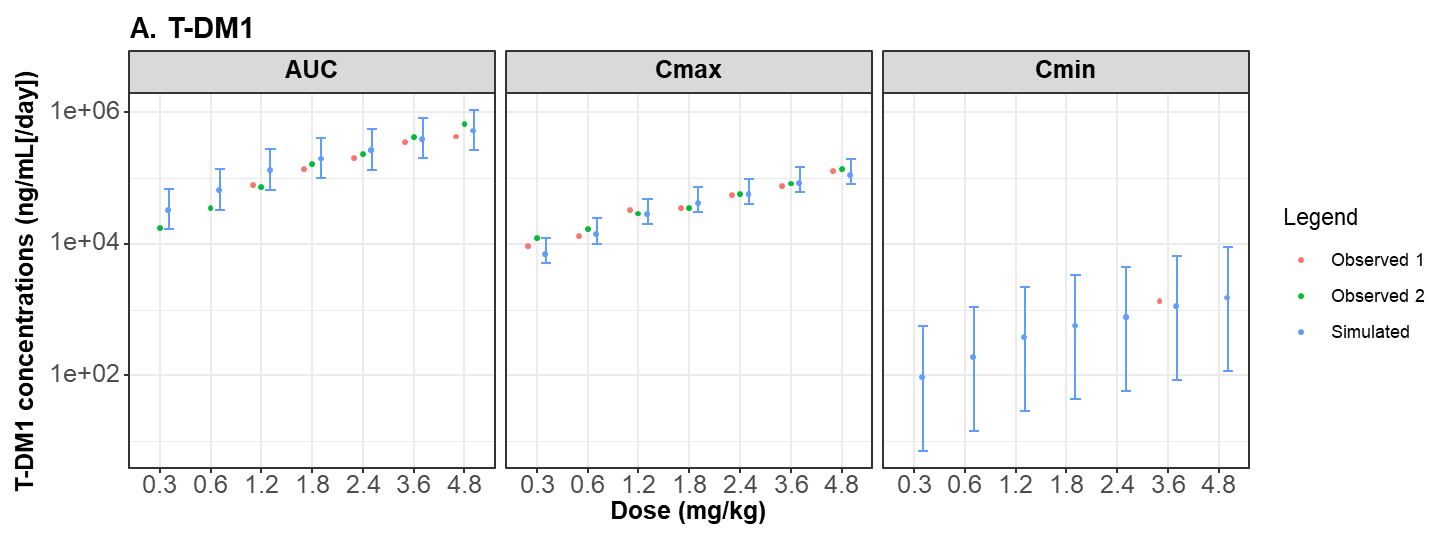


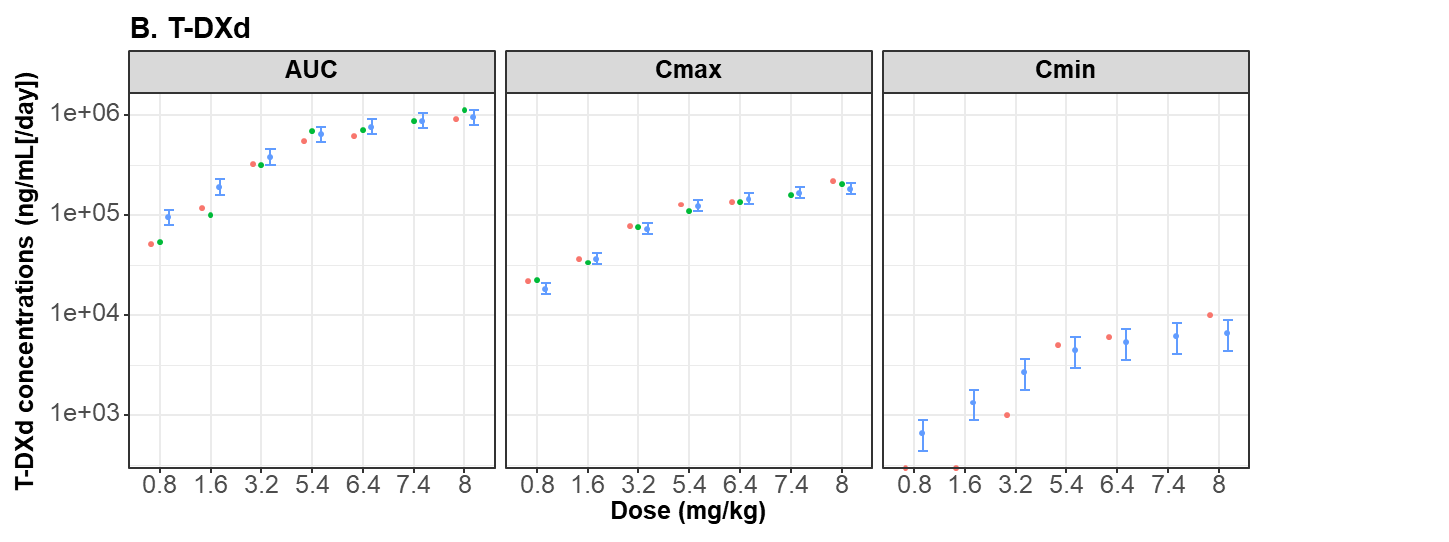


**Figure S5. Comparison of simulated and observed PK metrics (cycle 1) using Bayesian individual dynamic predictions.** **A.** Trastuzumab emtansine (T-DM1). **B.** Trastuzumab deruxtecan (T-DXd). The medians of the observed PK metrics were obtained from the reported summary metrics (‘Observed 1’, Tables S2 and S4) or computed from the digitized PK plots (‘Observed 2’, Figure S2) using the noncompartmental analysis R package PKNCA.^105^ Error bars on the simulated data represent 95% confidence intervals. AUC = area under the time-concentration curve, Cmax = maximum concentration, Cmin = minimum concentration, PK = pharmacokinetic.


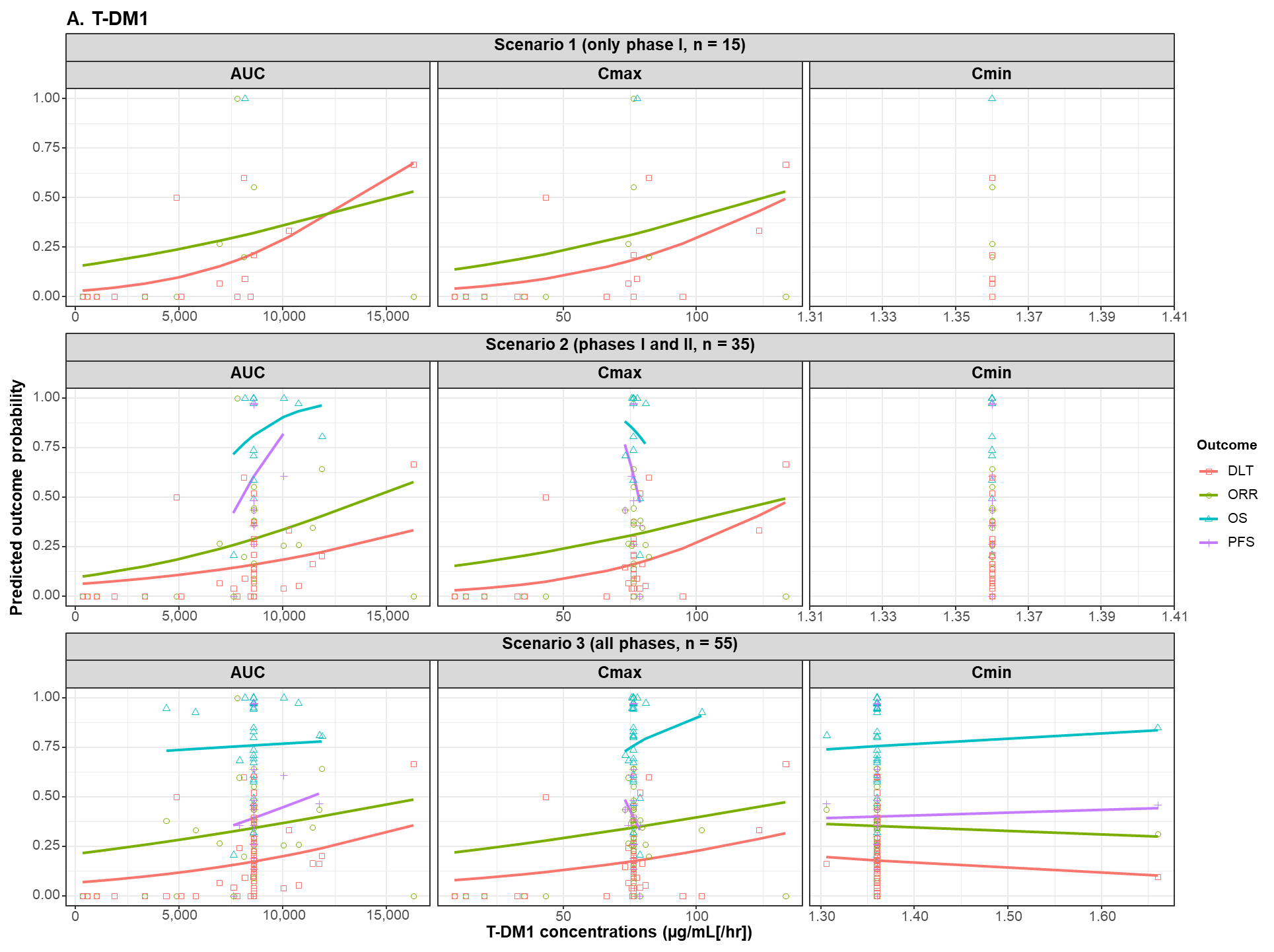


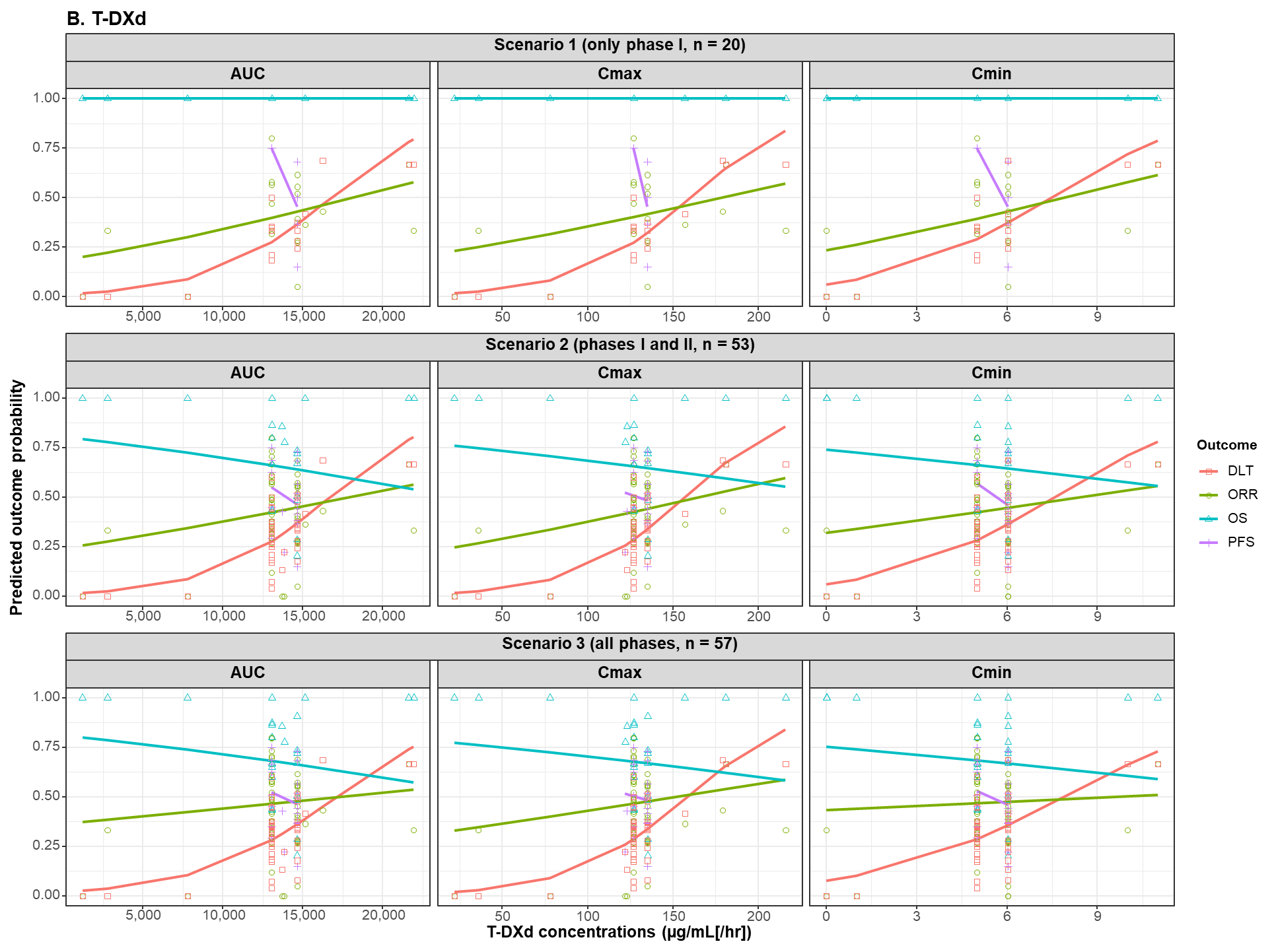


**Figure S6. Data used for Exposure-Response analysis for the Trastuzumab drug conjugates. A.** Trastuzumab emtansine (T-DM1). **B.** Trastuzumab deruxtecan (T-DXd). Logistic regression analysis was performed using the glm function from the R stats package, with the family set to 'quasibinomial', to fit the exposure-response curves. For visual clarity, the labels use µg/mL instead of the ng/mL used during analysis. AUC = area under the time-concentration curve, Cmax = maximum concentration, Cmin = minimum concentration, DLT = dose-limiting toxicity, ORR = objective response rate, OS = overall survival, PFS = progression-free survival.


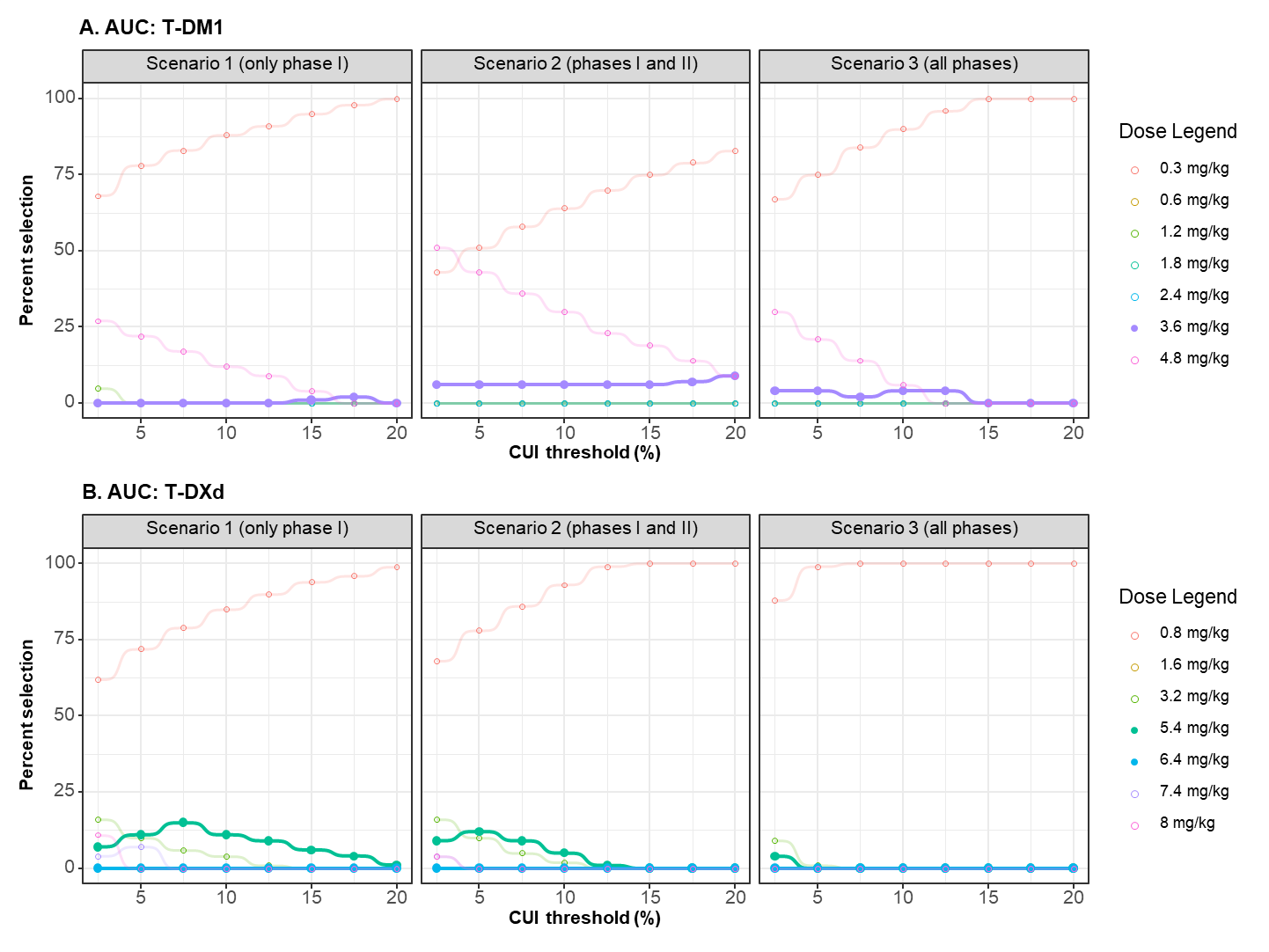


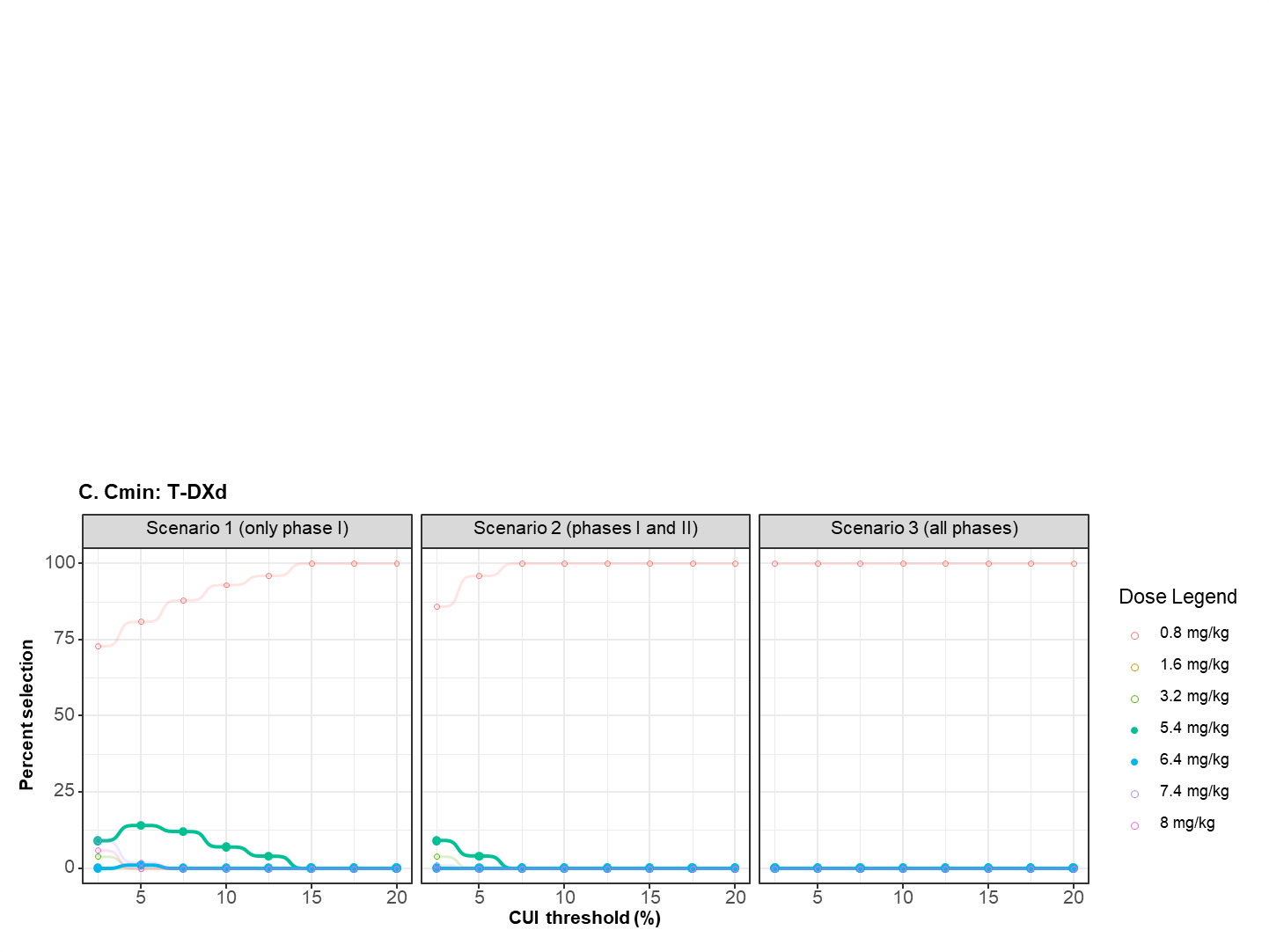


**Figure S7. Impact of CUI thresholds on dose selection for T-DM1 and T-DXd (AUC and Cmin as PK metrics).** This figure shows the effect of varying CUI thresholds (2.5% to 20%) on dose selection for T-DM1 with AUC as the PK metric (panel **A**), T-DXd with AUC as the PK metric (panel **B**) and T-DXd with Cmin as the PK metric (panel **C**) in three scenarios. T-DM1 with Cmin as the PK metric not shown since Cmin was reported for a single dose (3.6 mg/kg), resulting in insufficient data range for ER analysis. The CUI threshold was defined as the minimum percentage improvement in average CUI needed to select a higher dose. The approved doses of 3.6 mg/kg (T-DM1) and 5.4 and 6.4 mg/kg (T-DXd) are depicted using solid circles. AUC = area under the time-concentration curve (cycle 1), Cmin = minimum concentration, CUI = Clinical Utility Index, PK = pharmacokinetic, T-DM1 = Trastuzumab emtansine, T-DXd = Trastuzumab deruxtecan.

**Supplementary References**

1. Shen L, Sun X, Chen Z, et al. ADCdb: the database of antibody-drug conjugates. *Nucleic Acids Res*. Jan 5 2024;52(D1):D1097-D1109. doi:10.1093/nar/gkad831

2. Wang J, Song P, Schrieber S, et al. Exposure-response relationship of T-DM1: insight into dose optimization for patients with HER2-positive metastatic breast cancer. *Clinical pharmacology and therapeutics*. 2014;95(5):558-64. doi:<https://dx.doi.org/10.1038/clpt.2014.24>

3. Li C, Wang B, Chen S-C, et al. Exposure-response analyses of trastuzumab emtansine in patients with HER2-positive advanced breast cancer previously treated with trastuzumab and a taxane. *Cancer chemotherapy and pharmacology*. 2017;80(6):1079-1090. doi:<https://dx.doi.org/10.1007/s00280-017-3440-4>

4. Chen S-C, Quartino A, Polhamus D, et al. Population pharmacokinetics and exposure-response of trastuzumab emtansine in advanced breast cancer previously treated with >=2 HER2-targeted regimens. *British journal of clinical pharmacology*. 2017;83(12):2767-2777. doi:<https://dx.doi.org/10.1111/bcp.13381>

5. Krop IE, Beeram M, Modi S, et al. Phase I study of trastuzumab-DM1, an HER2 antibody-drug conjugate, given every 3 weeks to patients with HER2-positive metastatic breast cancer. *Journal of clinical oncology : official journal of the American Society of Clinical Oncology*. 2010;28(16):2698-704. doi:<https://dx.doi.org/10.1200/JCO.2009.26.2071>

6. Beeram M, Krop IE, Burris HA, et al. A phase 1 study of weekly dosing of trastuzumab emtansine (T-DM1) in patients with advanced human epidermal growth factor 2-positive breast cancer. *Cancer*. 2012;118(23):5733-40. doi:<https://dx.doi.org/10.1002/cncr.27622>

7. Burris HA, 3rd, Rugo HS, Vukelja SJ, et al. Phase II study of the antibody drug conjugate trastuzumab-DM1 for the treatment of human epidermal growth factor receptor 2 (HER2)-positive breast cancer after prior HER2-directed therapy. *Journal of clinical oncology : official journal of the American Society of Clinical Oncology*. 2011;29(4):398-405. Comment in: J Clin Oncol. 2011 Feb 1;29(4):351-4 PMID: 21172881 [<https://www.ncbi.nlm.nih.gov/pubmed/21172881>]. doi:<https://dx.doi.org/10.1200/JCO.2010.29.5865>

8. Girish S, Gupta M, Wang B, et al. Clinical pharmacology of trastuzumab emtansine (T-DM1): an antibody-drug conjugate in development for the treatment of HER2-positive cancer. *Cancer chemotherapy and pharmacology*. 2012;69(5):1229-40. doi:<https://dx.doi.org/10.1007/s00280-011-1817-3>

9. Krop IE, LoRusso P, Miller KD, et al. A phase II study of trastuzumab emtansine in patients with human epidermal growth factor receptor 2-positive metastatic breast cancer who were previously treated with trastuzumab, lapatinib, an anthracycline, a taxane, and capecitabine. *Journal of clinical oncology : official journal of the American Society of Clinical Oncology*. 2012;30(26):3234-41. doi:<https://dx.doi.org/10.1200/JCO.2011.40.5902>

10. Yamamoto H, Ando M, Aogi K, et al. Phase I and pharmacokinetic study of trastuzumab emtansine in Japanese patients with HER2-positive metastatic breast cancer. *Japanese journal of clinical oncology*. 2015;45(1):12-8. doi:<https://dx.doi.org/10.1093/jjco/hyu160>

11. Li C, Agarwal P, Gibiansky E, et al. A Phase I Pharmacokinetic Study of Trastuzumab Emtansine (T-DM1) in Patients with Human Epidermal Growth Factor Receptor 2-Positive Metastatic Breast Cancer and Normal or Reduced Hepatic Function. *Clinical pharmacokinetics*. 2017;56(9):1069-1080. doi:<https://dx.doi.org/10.1007/s40262-016-0496-y>

12. Chen S-C, Kagedal M, Gao Y, et al. Population pharmacokinetics of trastuzumab emtansine in previously treated patients with HER2-positive advanced gastric cancer (AGC). *Cancer chemotherapy and pharmacology*. 2017;80(6):1147-1159. Erratum in: Cancer Chemother Pharmacol. 2017 Nov 30;: PMID: 29189914 [<https://www.ncbi.nlm.nih.gov/pubmed/29189914>]. doi:<https://dx.doi.org/10.1007/s00280-017-3443-1>

13. Lu D, Li C, Riggs M, et al. Pharmacokinetics of trastuzumab emtansine (T-DM1) as a single agent or in combination with pertuzumab in HER2-positive breast cancer patients with recurrent or locally advanced metastatic breast cancer. *Cancer chemotherapy and pharmacology*. 2019;84(1):175-185. doi:<https://dx.doi.org/10.1007/s00280-019-03852-z>

14. Ji D, Shen W, Zhang J, et al. A phase I study of pharmacokinetics of trastuzumab emtansine in Chinese patients with locally advanced inoperable or metastatic human epidermal growth factor receptor 2-positive breast cancer who have received prior trastuzumab-based therapy. *Medicine*. 2020;99(44):e22886. doi:<https://dx.doi.org/10.1097/MD.0000000000022886>

15. de Vries EGE, Ruschoff J, Lolkema M, et al. Phase II study (KAMELEON) of single-agent T-DM1 in patients with HER2-positive advanced urothelial bladder cancer or pancreatic cancer/cholangiocarcinoma. *Cancer Med*. Jun 2023;12(11):12071-12083. doi:10.1002/cam4.5893

16. Li X, Wang Y, Hu W, Song Q, Ding L. Development and validation of pharmacokinetics assays for a novel HER2-targeting antibody-drug conjugate (SHR-A1201): Application to its dose-escalation pharmacokinetic study. *Journal of pharmaceutical and biomedical analysis*. 2024;240:115964. doi:<https://dx.doi.org/10.1016/j.jpba.2024.115964>

17. Li Y, Qi L, Wang Y, et al. Safety, tolerability, pharmacokinetics and immunogenicity of an antibody-drug conjugate (SHR-A1201) in patients with HER2-positive advanced breast cancer: an open, phase I dose-escalation study. *Anti-cancer drugs*. 2023;34(6):763-774. doi:<https://dx.doi.org/10.1097/CAD.0000000000001456>

18. Yin O, Xiong Y, Endo S, et al. Population Pharmacokinetics of Trastuzumab Deruxtecan in Patients With HER2-Positive Breast Cancer and Other Solid Tumors. *Clinical pharmacology and therapeutics*. 2021;109(5):1314-1325. doi:<https://dx.doi.org/10.1002/cpt.2096>

19. Doi T, Shitara K, Naito Y, et al. Safety, pharmacokinetics, and antitumour activity of trastuzumab deruxtecan (DS-8201), a HER2-targeting antibody-drug conjugate, in patients with advanced breast and gastric or gastro-oesophageal tumours: a phase 1 dose-escalation study. *The Lancet Oncology*. 2017;18(11):1512-1522. Comment in: Lancet Oncol. 2017 Nov;18(11):1433-1434 PMID: 29037986 [<https://www.ncbi.nlm.nih.gov/pubmed/29037986>]. doi:<https://dx.doi.org/10.1016/S1470-2045(17)30604-6>

20. Shimomura A, Takano T, Takahashi S, et al. Effect of Trastuzumab Deruxtecan on QT/QTc Interval and Pharmacokinetics in HER2-Positive or HER2-Low Metastatic/Unresectable Breast Cancer. *Clinical pharmacology and therapeutics*. 2023;113(1):160-169. doi:<https://dx.doi.org/10.1002/cpt.2757>

21. AstraZeneca. Data from: A Study of T-DXd for the Treatment of Solid Tumors Harboring HER2 Activating Mutations (DPT01). 2024. *ClinicalTrials.gov*.

22. Verma S, Miles D, Gianni L, et al. Trastuzumab emtansine for HER2-positive advanced breast cancer. *The New England journal of medicine*. 2012;367(19):1783-91. Erratum in: N Engl J Med. 2013 Jun 20;368(25):2442Comment in: N Engl J Med. 2012 Nov 8;367(19):1847-8 PMID: 23134386 [<https://www.ncbi.nlm.nih.gov/pubmed/23134386>]. doi:<https://dx.doi.org/10.1056/NEJMoa1209124>

23. Welslau M, Dieras V, Sohn J-H, et al. Patient-reported outcomes from EMILIA, a randomized phase 3 study of trastuzumab emtansine (T-DM1) versus capecitabine and lapatinib in human epidermal growth factor receptor 2-positive locally advanced or metastatic breast cancer. *Cancer*. 2014;120(5):642-51. doi:<https://dx.doi.org/10.1002/cncr.28465>

24. Krop IE, Lin NU, Blackwell K, et al. Trastuzumab emtansine (T-DM1) versus lapatinib plus capecitabine in patients with HER2-positive metastatic breast cancer and central nervous system metastases: a retrospective, exploratory analysis in EMILIA. *Annals of oncology : official journal of the European Society for Medical Oncology*. 2015;26(1):113-119. Comment in: Ann Oncol. 2015 May;26(5):1033-4 PMID: 25632067 [<https://www.ncbi.nlm.nih.gov/pubmed/25632067>]. doi:<https://dx.doi.org/10.1093/annonc/mdu486>

25. Baselga J, Lewis Phillips GD, Verma S, et al. Relationship between Tumor Biomarkers and Efficacy in EMILIA, a Phase III Study of Trastuzumab Emtansine in HER2-Positive Metastatic Breast Cancer. *Clinical cancer research : an official journal of the American Association for Cancer Research*. 2016;22(15):3755-63. Erratum in: Clin Cancer Res. 2018 Nov 1;24(21):5486 PMID: 30385658 [<https://www.ncbi.nlm.nih.gov/pubmed/30385658>]. doi:<https://dx.doi.org/10.1158/1078-0432.CCR-15-2499>

26. Li C, Wang B, Lu D, et al. Ethnic sensitivity assessment of the antibody-drug conjugate trastuzumab emtansine (T-DM1) in patients with HER2-positive locally advanced or metastatic breast cancer. *Cancer chemotherapy and pharmacology*. 2016;78(3):547-58. doi:<https://dx.doi.org/10.1007/s00280-016-3099-2>

27. Dieras V, Miles D, Verma S, et al. Trastuzumab emtansine versus capecitabine plus lapatinib in patients with previously treated HER2-positive advanced breast cancer (EMILIA): a descriptive analysis of final overall survival results from a randomised, open-label, phase 3 trial. *The Lancet Oncology*. 2017;18(6):732-742. Comment in: Lancet Oncol. 2017 Jun;18(6):696-697 PMID: 28526537 [<https://www.ncbi.nlm.nih.gov/pubmed/28526537>] Erratum in: Lancet Oncol. 2017 Aug;18(8):e433 PMID: 28759383 [<https://www.ncbi.nlm.nih.gov/pubmed/28759383>] Comment in: Nat Rev Clin Oncol. 2017 Nov;14(11):651-652 PMID: 28786416 [<https://www.ncbi.nlm.nih.gov/pubmed/28786416>] Erratum in: Lancet Oncol. 2018 Dec;19(12):e667 PMID: 30507428 [<https://www.ncbi.nlm.nih.gov/pubmed/30507428>]. doi:<https://dx.doi.org/10.1016/S1470-2045(17)30312-1>

28. Gupta M, Wang B, Carrothers TJ, et al. Effects of Trastuzumab Emtansine (T-DM1) on QT Interval and Safety of Pertuzumab Plus T-DM1 in Patients With Previously Treated Human Epidermal Growth Factor Receptor 2-Positive Metastatic Breast Cancer. *Clinical pharmacology in drug development*. 2013;2(1):11-24. doi:<https://dx.doi.org/10.1002/cpdd.9>

29. Hurvitz SA, Dirix L, Kocsis J, et al. Phase II randomized study of trastuzumab emtansine versus trastuzumab plus docetaxel in patients with human epidermal growth factor receptor 2-positive metastatic breast cancer. *Journal of clinical oncology : official journal of the American Society of Clinical Oncology*. 2013;31(9):1157-63. Erratum in: J Clin Oncol. 2013 Aug 10;31(23):2977Comment in: Future Oncol. 2013 Jul;9(7):955-7 PMID: 23837759 [<https://www.ncbi.nlm.nih.gov/pubmed/23837759>]. doi:<https://dx.doi.org/10.1200/JCO.2012.44.9694>

30. Perez EA, Hurvitz SA, Amler LC, et al. Relationship between HER2 expression and efficacy with first-line trastuzumab emtansine compared with trastuzumab plus docetaxel in TDM4450g: a randomized phase II study of patients with previously untreated HER2-positive metastatic breast cancer. *Breast cancer research : BCR*. 2014;16(3):R50. Comment in: Breast Cancer Res. 2014;16(6):478 PMID: 25472666 [<https://www.ncbi.nlm.nih.gov/pubmed/25472666>]. doi:<https://dx.doi.org/10.1186/bcr3661>

31. Krop IE, Kim SB, Gonzalez-Martin A, et al. Trastuzumab emtansine versus treatment of physician's choice for pretreated HER2-positive advanced breast cancer (TH3RESA): a randomised, open-label, phase 3 trial. *Lancet Oncol*. Jun 2014;15(7):689-99. doi:10.1016/S1470-2045(14)70178-0

32. Kim S-B, Wildiers H, Krop IE, et al. Relationship between tumor biomarkers and efficacy in TH3RESA, a phase III study of trastuzumab emtansine (T-DM1) vs. treatment of physician's choice in previously treated HER2-positive advanced breast cancer. *International journal of cancer*. 2016;139(10):2336-42. doi:<https://dx.doi.org/10.1002/ijc.30276>

33. Krop IE, Kim SB, Martin AG, et al. Trastuzumab emtansine versus treatment of physician's choice in patients with previously treated HER2-positive metastatic breast cancer (TH3RESA): final overall survival results from a randomised open-label phase 3 trial. *Lancet Oncol*. Jun 2017;18(6):743-754. doi:10.1016/S1470-2045(17)30313-3

34. Yardley DA, Krop IE, LoRusso PM, et al. Trastuzumab Emtansine (T-DM1) in Patients With HER2-Positive Metastatic Breast Cancer Previously Treated With Chemotherapy and 2 or More HER2-Targeted Agents: Results From the T-PAS Expanded Access Study. *Cancer journal (Sudbury, Mass)*. 2015;21(5):357-64. doi:<https://dx.doi.org/10.1097/PPO.0000000000000144>

35. Gebhart G, Lamberts LE, Wimana Z, et al. Molecular imaging as a tool to investigate heterogeneity of advanced HER2-positive breast cancer and to predict patient outcome under trastuzumab emtansine (T-DM1): the ZEPHIR trial. *Annals of oncology : official journal of the European Society for Medical Oncology*. 2016;27(4):619-24. Comment in: Ann Oncol. 2016 Apr;27(4):555-7 PMID: 26802154 [<https://www.ncbi.nlm.nih.gov/pubmed/26802154>] Comment in: Nature. 2017 Mar 29;543(7647):743-746 PMID: 28358075 [<https://www.ncbi.nlm.nih.gov/pubmed/28358075>]. doi:<https://dx.doi.org/10.1093/annonc/mdv577>

36. Jacot W, Pons E, Frenel J-S, et al. Efficacy and safety of trastuzumab emtansine (T-DM1) in patients with HER2-positive breast cancer with brain metastases. *Breast cancer research and treatment*. 2016;157(2):307-318. doi:<https://dx.doi.org/10.1007/s10549-016-3828-6>

37. Kashiwaba M, Ito Y, Takao S, et al. A multicenter Phase II study evaluating the efficacy, safety and pharmacokinetics of trastuzumab emtansine in Japanese patients with heavily pretreated HER2-positive locally recurrent or metastatic breast cancer. *Japanese journal of clinical oncology*. 2016;46(5):407-14. doi:<https://dx.doi.org/10.1093/jjco/hyw013>

38. Hoffmann-La Roche. Data from: A Study to Evaluate the Efficacy and Safety of Trastuzumab Emtansine Versus the Combination of Trastuzumab Plus Docetaxel in Patients With HER2-positive Breast Cancer. 2017. *ClinicalTrials.gov*.

39. Watanabe J, Ito Y, Saeki T, et al. Safety Evaluation of Trastuzumab Emtansine in Japanese Patients with HER2-Positive Advanced Breast Cancer. *In vivo (Athens, Greece)*. 2017;31(3):493-500. doi:<https://dx.doi.org/10.21873/invivo.11088>

40. Perez EA, Barrios C, Eiermann W, et al. Trastuzumab Emtansine With or Without Pertuzumab Versus Trastuzumab Plus Taxane for Human Epidermal Growth Factor Receptor 2-Positive, Advanced Breast Cancer: Primary Results From the Phase III MARIANNE Study. *Journal of clinical oncology : official journal of the American Society of Clinical Oncology*. 2017;35(2):141-148. Comment in: J Clin Oncol. 2017 Jan 10;35(2):127-130 PMID: 27918726 [<https://www.ncbi.nlm.nih.gov/pubmed/27918726>] Erratum in: J Clin Oncol. 2017 Jul 10;35(20):2342 PMID: 28679104 [<https://www.ncbi.nlm.nih.gov/pubmed/28679104>] Erratum in: J Clin Oncol. 2019 Feb 1;37(4):358 PMID: 30695670 [<https://www.ncbi.nlm.nih.gov/pubmed/30695670>]. doi:<https://dx.doi.org/10.1200/JCO.2016.67.4887>

41. Perez EA, Barrios C, Eiermann W, et al. Trastuzumab emtansine with or without pertuzumab versus trastuzumab with taxane for human epidermal growth factor receptor 2-positive advanced breast cancer: Final results from MARIANNE. *Cancer*. 2019;125(22):3974-3984. doi:<https://dx.doi.org/10.1002/cncr.32392>

42. Perez EA, de Haas SL, Eiermann W, et al. Relationship between tumor biomarkers and efficacy in MARIANNE, a phase III study of trastuzumab emtansine +/- pertuzumab versus trastuzumab plus taxane in HER2-positive advanced breast cancer. *BMC cancer*. 2019;19(1):517. Erratum in: BMC Cancer. 2019 Jun 24;19(1):620 PMID: 31234810 [<https://www.ncbi.nlm.nih.gov/pubmed/31234810>]. doi:<https://dx.doi.org/10.1186/s12885-019-5687-0>

43. Fabi A, De Laurentiis M, Caruso M, et al. Efficacy and safety of T-DM1 in the 'common-practice' of HER2+ advanced breast cancer setting: a multicenter study. *Oncotarget*. 2017;8(38):64481-64489. doi:<https://dx.doi.org/10.18632/oncotarget.16373>

44. Fabi A, Giannarelli D, Moscetti L, et al. Ado-trastuzumab emtansine (T-DM1) in HER2+ advanced breast cancer patients: does pretreatment with pertuzumab matter? *Future Oncol*. Dec 2017;13(30):2791-2797. doi:10.2217/fon-2017-0336

45. Fabi A, Alesini D, Valle E, et al. T-DM1 and brain metastases: Clinical outcome in HER2-positive metastatic breast cancer. *Breast (Edinburgh, Scotland)*. 2018;41:137-143. doi:<https://dx.doi.org/10.1016/j.breast.2018.07.004>

46. Harbeck N, Gluz O, Christgen M, et al. De-Escalation Strategies in Human Epidermal Growth Factor Receptor 2 (HER2)-Positive Early Breast Cancer (BC): Final Analysis of the West German Study Group Adjuvant Dynamic Marker-Adjusted Personalized Therapy Trial Optimizing Risk Assessment and Therapy Response Prediction in Early BC HER2- and Hormone Receptor-Positive Phase II Randomized Trial-Efficacy, Safety, and Predictive Markers for 12 Weeks of Neoadjuvant Trastuzumab Emtansine With or Without Endocrine Therapy (ET) Versus Trastuzumab Plus ET. *Journal of clinical oncology : official journal of the American Society of Clinical Oncology*. 2017;35(26):3046-3054. doi:<https://dx.doi.org/10.1200/JCO.2016.71.9815>

47. Harbeck N, Nitz UA, Christgen M, et al. De-Escalated Neoadjuvant Trastuzumab-Emtansine With or Without Endocrine Therapy Versus Trastuzumab With Endocrine Therapy in HR+/HER2+ Early Breast Cancer: 5-Year Survival in the WSG-ADAPT-TP Trial. *Journal of clinical oncology : official journal of the American Society of Clinical Oncology*. 2023;41(22):3796-3804. doi:<https://dx.doi.org/10.1200/JCO.22.01816>

48. Li BT, Shen R, Buonocore D, et al. Ado-Trastuzumab Emtansine for Patients With HER2-Mutant Lung Cancers: Results From a Phase II Basket Trial. *J Clin Oncol*. Aug 20 2018;36(24):2532-2537. doi:10.1200/JCO.2018.77.9777

49. Li BT, Michelini F, Misale S, et al. HER2-Mediated Internalization of Cytotoxic Agents in ERBB2 Amplified or Mutant Lung Cancers. *Cancer discovery*. 2020;10(5):674-687. Comment in: Cancer Discov. 2020 May;10(5):643-645 PMID: 32357968 [<https://www.ncbi.nlm.nih.gov/pubmed/32357968>]. doi:<https://dx.doi.org/10.1158/2159-8290.CD-20-0215>

50. Hotta K, Aoe K, Kozuki T, et al. A Phase II Study of Trastuzumab Emtansine in HER2-Positive Non-Small Cell Lung Cancer. *Journal of thoracic oncology : official publication of the International Association for the Study of Lung Cancer*. 2018;13(2):273-279. doi:<https://dx.doi.org/10.1016/j.jtho.2017.10.032>

51. Wildiers H, Tryfonidis K, Dal Lago L, et al. Pertuzumab and trastuzumab with or without metronomic chemotherapy for older patients with HER2-positive metastatic breast cancer (EORTC 75111-10114): an open-label, randomised, phase 2 trial from the Elderly Task Force/Breast Cancer Group. *The Lancet Oncology*. 2018;19(3):323-336. Comment in: Lancet Oncol. 2018 Mar;19(3):271-272 PMID: 29433964 [<https://www.ncbi.nlm.nih.gov/pubmed/29433964>] Comment in: Nat Rev Clin Oncol. 2018 May;15(5):266 PMID: 29485134 [<https://www.ncbi.nlm.nih.gov/pubmed/29485134>]. doi:<https://dx.doi.org/10.1016/S1470-2045(18)30083-4>

52. Wildiers H, Meyskens T, Marreaud S, et al. Long term outcome data from the EORTC 75111-10114 ETF/BCG randomized phase II study: Pertuzumab and trastuzumab with or without metronomic chemotherapy for older patients with HER2-positive metastatic breast cancer, followed by T-DM1 after progression. *Breast*. Aug 2022;64:100-111. doi:10.1016/j.breast.2022.05.004

53. Yeo W, Luk MY, Soong IS, et al. Efficacy and tolerability of trastuzumab emtansine in advanced human epidermal growth factor receptor 2-positive breast cancer. *Hong Kong medical journal = Xianggang yi xue za zhi*. 2018;24(1):56-62. Comment in: Hong Kong Med J. 2018 Feb;24(1):6-8 PMID: 29424344 [<https://www.ncbi.nlm.nih.gov/pubmed/29424344>]. doi:<https://dx.doi.org/10.12809/hkmj176808>

54. Jacot W, Cottu P, Berger F, et al. Actionability of HER2-amplified circulating tumor cells in HER2-negative metastatic breast cancer: the CirCe T-DM1 trial. *Breast cancer research : BCR*. 2019;21(1):121. doi:<https://dx.doi.org/10.1186/s13058-019-1215-z>

55. Lynce F, Barac A, Geng X, et al. Prospective evaluation of the cardiac safety of HER2-targeted therapies in patients with HER2-positive breast cancer and compromised heart function: the SAFE-HEaRt study. *Breast cancer research and treatment*. 2019;175(3):595-603. doi:<https://dx.doi.org/10.1007/s10549-019-05191-2>

56. Montemurro F, Ellis P, Anton A, et al. Safety of trastuzumab emtansine (T-DM1) in patients with HER2-positive advanced breast cancer: Primary results from the KAMILLA study cohort 1. *European journal of cancer (Oxford, England : 1990)*. 2019;109:92-102. Comment in: Eur J Cancer. 2019 Aug;117:1-4 PMID: 31229945 [<https://www.ncbi.nlm.nih.gov/pubmed/31229945>]. doi:<https://dx.doi.org/10.1016/j.ejca.2018.12.022>

57. Montemurro F, Delaloge S, Barrios CH, et al. Trastuzumab emtansine (T-DM1) in patients with HER2-positive metastatic breast cancer and brain metastases: exploratory final analysis of cohort 1 from KAMILLA, a single-arm phase IIIb clinical trial. *Annals of oncology : official journal of the European Society for Medical Oncology*. 2020;31(10):1350-1358. Comment in: Ann Oncol. 2020 Oct;31(10):1279-1281 PMID: 32652113 [<https://www.ncbi.nlm.nih.gov/pubmed/32652113>]. doi:<https://dx.doi.org/10.1016/j.annonc.2020.06.020>

58. Wuerstlein R, Ellis P, Montemurro F, et al. Final results of the global and Asia cohorts of KAMILLA, a phase IIIB safety trial of trastuzumab emtansine in patients with HER2-positive advanced breast cancer. *ESMO open*. 2022;7(5):100561. doi:<https://dx.doi.org/10.1016/j.esmoop.2022.100561>

59. von Minckwitz G, Huang C-S, Mano MS, et al. Trastuzumab Emtansine for Residual Invasive HER2-Positive Breast Cancer. *The New England journal of medicine*. 2019;380(7):617-628. Comment in: Cancer Discov. 2019 Feb;9(2):158-159 PMID: 30541774 [<https://www.ncbi.nlm.nih.gov/pubmed/30541774>] Comment in: N Engl J Med. 2019 Feb 14;380(7):676-677 PMID: 30763184 [<https://www.ncbi.nlm.nih.gov/pubmed/30763184>] Comment in: Natl Med J India. 2020 May-Jun;33(3):158-159 PMID: 33904420 [<https://www.ncbi.nlm.nih.gov/pubmed/33904420>] Comment in: Int J Radiat Oncol Biol Phys. 2022 Nov 1;114(3):377-381 PMID: 36152640 [<https://www.ncbi.nlm.nih.gov/pubmed/36152640>]. doi:<https://dx.doi.org/10.1056/NEJMoa1814017>

60. Mamounas EP, Untch M, Mano MS, et al. Adjuvant T-DM1 versus trastuzumab in patients with residual invasive disease after neoadjuvant therapy for HER2-positive breast cancer: subgroup analyses from KATHERINE. *Annals of oncology : official journal of the European Society for Medical Oncology*. 2021;32(8):1005-1014. Comment in: Ann Oncol. 2021 Sep;32(9):1191 PMID: 34023398 [<https://www.ncbi.nlm.nih.gov/pubmed/34023398>] Comment in: Ann Oncol. 2021 Sep;32(9):1191-1192 PMID: 34058348 [<https://www.ncbi.nlm.nih.gov/pubmed/34058348>]. doi:<https://dx.doi.org/10.1016/j.annonc.2021.04.011>

61. Huang C-S, Yang Y, Kwong A, et al. Trastuzumab emtansine (T-DM1) versus trastuzumab in Chinese patients with residual invasive disease after neoadjuvant chemotherapy and HER2-targeted therapy for HER2-positive breast cancer in the phase 3 KATHERINE study. *Breast cancer research and treatment*. 2021;187(3):759-768. doi:<https://dx.doi.org/10.1007/s10549-021-06166-y>

62. Peters S, Stahel R, Bubendorf L, et al. Trastuzumab Emtansine (T-DM1) in Patients with Previously Treated HER2-Overexpressing Metastatic Non-Small Cell Lung Cancer: Efficacy, Safety, and Biomarkers. *Clinical cancer research : an official journal of the American Association for Cancer Research*. 2019;25(1):64-72. doi:<https://dx.doi.org/10.1158/1078-0432.CCR-18-1590>

63. Battisti NML, Rogerson F, Lee K, et al. Safety and efficacy of T-DM1 in patients with advanced HER2-positive breast cancer The Royal Marsden experience. *Cancer treatment and research communications*. 2020;24:100188. doi:<https://dx.doi.org/10.1016/j.ctarc.2020.100188>

64. Cortes J, Dieras V, Lorenzen S, et al. Efficacy and Safety of Trastuzumab Emtansine Plus Capecitabine vs Trastuzumab Emtansine Alone in Patients With Previously Treated ERBB2 (HER2)-Positive Metastatic Breast Cancer: A Phase 1 and Randomized Phase 2 Trial. *JAMA oncology*. 2020;6(8):1203-1209. doi:<https://dx.doi.org/10.1001/jamaoncol.2020.1796>

65. Emens LA, Esteva FJ, Beresford M, et al. Trastuzumab emtansine plus atezolizumab versus trastuzumab emtansine plus placebo in previously treated, HER2-positive advanced breast cancer (KATE2): a phase 2, multicentre, randomised, double-blind trial. *Lancet Oncol*. Oct 2020;21(10):1283-1295. doi:10.1016/S1470-2045(20)30465-4

66. Hoffmann-La Roche. Data from: A Study of Trastuzumab Emtansine in Indian Patients With Human Epidermal Growth Factor Receptor 2 (HER2)-Positive Unresectable Locally Advanced or Metastatic Breast Cancer Who Have Received Prior Treatment With Trastuzumab and a Taxane. 2021. *ClinicalTrials.gov*.

67. Tolaney SM, Tayob N, Dang C, et al. Adjuvant Trastuzumab Emtansine Versus Paclitaxel in Combination With Trastuzumab for Stage I HER2-Positive Breast Cancer (ATEMPT): A Randomized Clinical Trial. *J Clin Oncol*. Jul 20 2021;39(21):2375-2385. doi:10.1200/JCO.20.03398

68. Acibuca A, Sezer A, Yilmaz M, et al. Cardiotoxicity of trastuzumab emtansine (T-DM1): a single-center experience. *The Journal of international medical research*. 2021;49(12):3000605211053755. doi:<https://dx.doi.org/10.1177/03000605211053755>

69. Hatschek T, Foukakis T, Bjohle J, et al. Neoadjuvant Trastuzumab, Pertuzumab, and Docetaxel vs Trastuzumab Emtansine in Patients With ERBB2-Positive Breast Cancer: A Phase 2 Randomized Clinical Trial. *JAMA oncology*. 2021;7(9):1360-1367. Erratum in: JAMA Oncol. 2021 Sep 1;7(9):1405-1406 PMID: 34383001 [<https://www.ncbi.nlm.nih.gov/pubmed/34383001>]. doi:<https://dx.doi.org/10.1001/jamaoncol.2021.1932>

70. Bahceci A, Paydas S, Ak N, et al. Efficacy and Safety of Trastuzumab Emtansine in Her2 Positive Metastatic Breast Cancer: Real-World Experience. *Cancer investigation*. 2021;39(6-7):473-481. doi:<https://dx.doi.org/10.1080/07357907.2021.1933011>

71. Tataroglu Ozyukseler D, Basak M, Ay S, et al. Prognostic factors of ado-trastuzumab emtansine treatment in patients with metastatic HER-2 positive breast cancer. *Journal of oncology pharmacy practice : official publication of the International Society of Oncology Pharmacy Practitioners*. 2021;27(3):547-554. doi:<https://dx.doi.org/10.1177/1078155220924088>

72. Iwama E, Zenke Y, Sugawara S, et al. Trastuzumab emtansine for patients with non-small cell lung cancer positive for human epidermal growth factor receptor 2 exon-20 insertion mutations. *European journal of cancer (Oxford, England : 1990)*. 2022;162:99-106. doi:<https://dx.doi.org/10.1016/j.ejca.2021.11.021>

73. Chiradoni Thungappa S, Maksud T, Raut N, et al. Comparison of the Efficacy, Safety, Pharmacokinetic and Immunogenicity of UJVIRA (ZRC-3256, Trastuzumab Emtansine) With the Kadcyla (Trastuzumab Emtansine) in the Treatment of HER2-Positive Metastatic Breast Cancer: A Randomized, Open-Label, Multicenter Study in India. *Clinical breast cancer*. 2022;22(4):300-307. doi:<https://dx.doi.org/10.1016/j.clbc.2021.11.006>

74. Cortes J, Kim S-B, Chung W-P, et al. Trastuzumab Deruxtecan versus Trastuzumab Emtansine for Breast Cancer. *The New England journal of medicine*. 2022;386(12):1143-1154. Comment in: Nat Rev Clin Oncol. 2022 May;19(5):283 PMID: 35361895 [<https://www.ncbi.nlm.nih.gov/pubmed/35361895>] Comment in: N Engl J Med. 2022 Jun 16;386(24):2346-2347 PMID: 35704495 [<https://www.ncbi.nlm.nih.gov/pubmed/35704495>] Comment in: Cell Rep Med. 2022 Jun 21;3(6):100668 PMID: 35732147 [<https://www.ncbi.nlm.nih.gov/pubmed/35732147>]. doi:<https://dx.doi.org/10.1056/NEJMoa2115022>

75. Curigliano G, Dunton K, Rosenlund M, et al. Patient-reported outcomes and hospitalization data in patients with HER2-positive metastatic breast cancer receiving trastuzumab deruxtecan or trastuzumab emtansine in the phase III DESTINY-Breast03 study. *Annals of oncology : official journal of the European Society for Medical Oncology*. 2023;34(7):569-577. doi:<https://dx.doi.org/10.1016/j.annonc.2023.04.516>

76. Hurvitz SA, Hegg R, Chung W-P, et al. Trastuzumab deruxtecan versus trastuzumab emtansine in patients with HER2-positive metastatic breast cancer: updated results from DESTINY-Breast03, a randomised, open-label, phase 3 trial. *Lancet (London, England)*. 2023;401(10371):105-117. Comment in: Lancet. 2023 Jan 14;401(10371):80-81 PMID: 36495878 [<https://www.ncbi.nlm.nih.gov/pubmed/36495878>] Erratum in: Lancet. 2023 Feb 18;401(10376):556 PMID: 36803433 [<https://www.ncbi.nlm.nih.gov/pubmed/36803433>] Comment in: Lancet. 2023 May 20;401(10389):1652 PMID: 37210114 [<https://www.ncbi.nlm.nih.gov/pubmed/37210114>] Comment in: Lancet. 2023 May 20;401(10389):1652-1653 PMID: 37210115 [<https://www.ncbi.nlm.nih.gov/pubmed/37210115>] Comment in: Lancet. 2023 May 20;401(10389):1653 PMID: 37210116 [<https://www.ncbi.nlm.nih.gov/pubmed/37210116>]. doi:<https://dx.doi.org/10.1016/S0140-6736(22)02420-5>

77. Ji C, Li F, Yuan Y, et al. Novel Anti-HER2 Antibody-Drug Conjugates Versus T-DM1 for HER2-Positive Metastatic Breast Cancer After Tyrosine Kinase Inhibitors Treatment. *The oncologist*. 2023;28(10):e859-e866. doi:<https://dx.doi.org/10.1093/oncolo/oyad127>

78. Modi S, Saura C, Yamashita T, et al. Trastuzumab Deruxtecan in Previously Treated HER2-Positive Breast Cancer. *The New England journal of medicine*. 2020;382(7):610-621. Comment in: Cancer Discov. 2020 Feb;10(2):167 PMID: 31843763 [<https://www.ncbi.nlm.nih.gov/pubmed/31843763>] Comment in: Nat Rev Clin Oncol. 2020 Mar;17(3):133 PMID: 31900443 [<https://www.ncbi.nlm.nih.gov/pubmed/31900443>] Comment in: N Engl J Med. 2020 Feb 13;382(7):669-671 PMID: 32053305 [<https://www.ncbi.nlm.nih.gov/pubmed/32053305>] Comment in: Future Oncol. 2021 Sep 1;17(26):3415-3423 PMID: 34263665 [<https://www.ncbi.nlm.nih.gov/pubmed/34263665>]. doi:<https://dx.doi.org/10.1056/NEJMoa1914510>

79. Jerusalem G, Park YH, Yamashita T, et al. Trastuzumab Deruxtecan in HER2-Positive Metastatic Breast Cancer Patients with Brain Metastases: A DESTINY-Breast01 Subgroup Analysis. *Cancer discovery*. 2022;12(12):2754-2762. doi:<https://dx.doi.org/10.1158/2159-8290.CD-22-0837>

80. Saura C, Modi S, Krop I, et al. Trastuzumab deruxtecan in previously treated patients with HER2-positive metastatic breast cancer: updated survival results from a phase II trial (DESTINY-Breast01). *Annals of oncology : official journal of the European Society for Medical Oncology*. 2024;35(3):302-307. doi:<https://dx.doi.org/10.1016/j.annonc.2023.12.001>

81. Li BT, Smit EF, Goto Y, et al. Trastuzumab Deruxtecan in HER2-Mutant Non-Small-Cell Lung Cancer. *The New England journal of medicine*. 2022;386(3):241-251. Comment in: Nat Rev Clin Oncol. 2021 Dec;18(12):748 PMID: 34611335 [<https://www.ncbi.nlm.nih.gov/pubmed/34611335>] Comment in: N Engl J Med. 2022 Jan 20;386(3):286-289 PMID: 35045232 [<https://www.ncbi.nlm.nih.gov/pubmed/35045232>] Comment in: N Engl J Med. 2022 May 5;386(18):1769-1770 PMID: 35507494 [<https://www.ncbi.nlm.nih.gov/pubmed/35507494>] Comment in: N Engl J Med. 2022 May 5;386(18):1770 PMID: 35507495 [<https://www.ncbi.nlm.nih.gov/pubmed/35507495>] Comment in: Am J Respir Crit Care Med. 2024 Mar 15;209(6):748-750 PMID: 38190703 [<https://www.ncbi.nlm.nih.gov/pubmed/38190703>]. doi:<https://dx.doi.org/10.1056/NEJMoa2112431>

82. Smit EF, Felip E, Uprety D, et al. Trastuzumab deruxtecan in patients with metastatic non-small-cell lung cancer (DESTINY-Lung01): primary results of the HER2-overexpressing cohorts from a single-arm, phase 2 trial. *The Lancet Oncology*. 2024;25(4):439-454. doi:<https://dx.doi.org/10.1016/S1470-2045(24)00064-0>

83. Siena S, Di Bartolomeo M, Raghav K, et al. Trastuzumab deruxtecan (DS-8201) in patients with HER2-expressing metastatic colorectal cancer (DESTINY-CRC01): a multicentre, open-label, phase 2 trial. *The Lancet Oncology*. 2021;22(6):779-789. Comment in: Lancet Oncol. 2021 Jun;22(6):739-741 PMID: 33961794 [<https://www.ncbi.nlm.nih.gov/pubmed/33961794>]. doi:<https://dx.doi.org/10.1016/S1470-2045(21)00086-3>

84. Yoshino T, Di Bartolomeo M, Raghav K, et al. Final results of DESTINY-CRC01 investigating trastuzumab deruxtecan in patients with HER2-expressing metastatic colorectal cancer. *Nature communications*. 2023;14(1):3332. doi:<https://dx.doi.org/10.1038/s41467-023-38032-4>

85. Andre F, Hee Park Y, Kim S-B, et al. Trastuzumab deruxtecan versus treatment of physician's choice in patients with HER2-positive metastatic breast cancer (DESTINY-Breast02): a randomised, open-label, multicentre, phase 3 trial. *Lancet (London, England)*. 2023;401(10390):1773-1785. Comment in: Lancet. 2023 May 27;401(10390):1746-1747 PMID: 37086743 [<https://www.ncbi.nlm.nih.gov/pubmed/37086743>] Comment in: Nat Rev Clin Oncol. 2023 Jul;20(7):426 PMID: 37173585 [<https://www.ncbi.nlm.nih.gov/pubmed/37173585>] Erratum in: Lancet. 2023 Dec 9;402(10418):2196 PMID: 38070948 [<https://www.ncbi.nlm.nih.gov/pubmed/38070948>] Comment in: Transl Cancer Res. 2023 Nov 30;12(11):2979-2983 PMID: 38130308 [<https://www.ncbi.nlm.nih.gov/pubmed/38130308>] Erratum in: Lancet. 2024 Mar 9;403(10430):912 PMID: 38460989 [<https://www.ncbi.nlm.nih.gov/pubmed/38460989>]. doi:<https://dx.doi.org/10.1016/S0140-6736(23)00725-0>

86. Raghav K, Siena S, Takashima A, et al. Trastuzumab deruxtecan (T-DXd) in patients (pts) with HER2-overexpressing/amplified (HER2+) metastatic colorectal cancer (mCRC): Primary results from the multicenter, randomized, phase 2 DESTINY-CRC02 study. *Journal of clinical oncology : official journal of the American Society of Clinical Oncology*. 2023;41(16):3501. doi:<https://doi.org/10.1200/JCO.2023.41.16_suppl.3501>

87. Shitara K, Bang Y-J, Iwasa S, et al. Trastuzumab Deruxtecan in Previously Treated HER2-Positive Gastric Cancer. *The New England journal of medicine*. 2020;382(25):2419-2430. Comment in: Nat Rev Clin Oncol. 2020 Sep;17(9):521 PMID: 32533051 [<https://www.ncbi.nlm.nih.gov/pubmed/32533051>] Comment in: Cancer Cell. 2020 Sep 14;38(3):317-319 PMID: 32857948 [<https://www.ncbi.nlm.nih.gov/pubmed/32857948>]. doi:<https://dx.doi.org/10.1056/NEJMoa2004413>

88. Yamaguchi K, Bang Y-J, Iwasa S, et al. Trastuzumab Deruxtecan in Anti-Human Epidermal Growth Factor Receptor 2 Treatment-Naive Patients With Human Epidermal Growth Factor Receptor 2-Low Gastric or Gastroesophageal Junction Adenocarcinoma: Exploratory Cohort Results in a Phase II Trial. *Journal of clinical oncology : official journal of the American Society of Clinical Oncology*. 2023;41(4):816-825. doi:<https://dx.doi.org/10.1200/JCO.22.00575>

89. Meric-Bernstam F, Makker V, Oaknin A, et al. Efficacy and Safety of Trastuzumab Deruxtecan in Patients With HER2-Expressing Solid Tumors: Primary Results From the DESTINY-PanTumor02 Phase II Trial. *Journal of clinical oncology : official journal of the American Society of Clinical Oncology*. 2024;42(1):47-58. Comment in: Cancer Discov. 2023 Dec 12;13(12):OF10 PMID: 37888905 [<https://www.ncbi.nlm.nih.gov/pubmed/37888905>]. doi:<https://dx.doi.org/10.1200/JCO.23.02005>

90. Mosele F, Deluche E, Lusque A, et al. Trastuzumab deruxtecan in metastatic breast cancer with variable HER2 expression: the phase 2 DAISY trial. *Nature medicine*. 2023;29(8):2110-2120. doi:<https://dx.doi.org/10.1038/s41591-023-02478-2>

91. Bartsch R, Berghoff AS, Furtner J, et al. Trastuzumab deruxtecan in HER2-positive breast cancer with brain metastases: a single-arm, phase 2 trial. *Nature medicine*. 2022;28(9):1840-1847. doi:<https://dx.doi.org/10.1038/s41591-022-01935-8>

92. Shitara K, Iwata H, Takahashi S, et al. Trastuzumab deruxtecan (DS-8201a) in patients with advanced HER2-positive gastric cancer: a dose-expansion, phase 1 study. *The Lancet Oncology*. 2019;20(6):827-836. Comment in: Lancet Oncol. 2019 Jun;20(6):748-750 PMID: 31047805 [<https://www.ncbi.nlm.nih.gov/pubmed/31047805>] Erratum in: Lancet Oncol. 2019 May 10;: PMID: 31085052 [<https://www.ncbi.nlm.nih.gov/pubmed/31085052>]. doi:<https://dx.doi.org/10.1016/S1470-2045(19)30088-9>

93. Tamura K, Tsurutani J, Takahashi S, et al. Trastuzumab deruxtecan (DS-8201a) in patients with advanced HER2-positive breast cancer previously treated with trastuzumab emtansine: a dose-expansion, phase 1 study. *The Lancet Oncology*. 2019;20(6):816-826. Comment in: Lancet Oncol. 2019 Jun;20(6):748-750 PMID: 31047805 [<https://www.ncbi.nlm.nih.gov/pubmed/31047805>] Erratum in: Lancet Oncol. 2019 May 10;: PMID: 31085051 [<https://www.ncbi.nlm.nih.gov/pubmed/31085051>]. doi:<https://dx.doi.org/10.1016/S1470-2045(19)30097-X>

94. Tsurutani J, Iwata H, Krop I, et al. Targeting HER2 with Trastuzumab Deruxtecan: A Dose-Expansion, Phase I Study in Multiple Advanced Solid Tumors. *Cancer discovery*. 2020;10(5):688-701. Erratum in: Cancer Discov. 2020 Jul;10(7):1078 PMID: 32611736 [<https://www.ncbi.nlm.nih.gov/pubmed/32611736>]. doi:<https://dx.doi.org/10.1158/2159-8290.CD-19-1014>

95. Modi S, Park H, Murthy RK, et al. Antitumor Activity and Safety of Trastuzumab Deruxtecan in Patients With HER2-Low-Expressing Advanced Breast Cancer: Results From a Phase Ib Study. *Journal of clinical oncology : official journal of the American Society of Clinical Oncology*. 2020;38(17):1887-1896. Comment in: Cancer Discov. 2020 Apr;10(4):488 PMID: 32111601 [<https://www.ncbi.nlm.nih.gov/pubmed/32111601>] Comment in: J Clin Oncol. 2020 Oct 1;38(28):3350-3351 PMID: 32658628 [<https://www.ncbi.nlm.nih.gov/pubmed/32658628>] Comment in: J Clin Oncol. 2020 Oct 1;38(28):3351-3352 PMID: 32658630 [<https://www.ncbi.nlm.nih.gov/pubmed/32658630>]. doi:<https://dx.doi.org/10.1200/JCO.19.02318>

96. Takahashi S, Karayama M, Takahashi M, et al. Pharmacokinetics, Safety, and Efficacy of Trastuzumab Deruxtecan with Concomitant Ritonavir or Itraconazole in Patients with HER2-Expressing Advanced Solid Tumors. *Clinical cancer research : an official journal of the American Association for Cancer Research*. 2021;27(21):5771-5780. doi:<https://dx.doi.org/10.1158/1078-0432.CCR-21-1560>

97. Modi S, Jacot W, Yamashita T, et al. Trastuzumab Deruxtecan in Previously Treated HER2-Low Advanced Breast Cancer. *N Engl J Med*. Jul 7 2022;387(1):9-20. doi:10.1056/NEJMoa2203690

98. Narayan P, Dilawari A, Osgood C, et al. US Food and Drug Administration Approval Summary: Fam-Trastuzumab Deruxtecan-nxki for Human Epidermal Growth Factor Receptor 2-Low Unresectable or Metastatic Breast Cancer. *Journal of clinical oncology : official journal of the American Society of Clinical Oncology*. 2023;41(11):2108-2116. doi:<https://dx.doi.org/10.1200/JCO.22.02447>

99. Goto K, Goto Y, Kubo T, et al. Trastuzumab Deruxtecan in Patients With HER2-Mutant Metastatic Non-Small-Cell Lung Cancer: Primary Results From the Randomized, Phase II DESTINY-Lung02 Trial. *Journal of clinical oncology : official journal of the American Society of Clinical Oncology*. 2023;41(31):4852-4863. Comment in: J Clin Oncol. 2023 Nov 1;41(31):4849-4851 PMID: 37694345 [<https://www.ncbi.nlm.nih.gov/pubmed/37694345>] Erratum in: J Clin Oncol. 2023 Dec 19;:JCO2302574 PMID: 38113428 [<https://www.ncbi.nlm.nih.gov/pubmed/38113428>]. doi:<https://dx.doi.org/10.1200/JCO.23.01361>

100. Van Cutsem E, di Bartolomeo M, Smyth E, et al. Trastuzumab deruxtecan in patients in the USA and Europe with HER2-positive advanced gastric or gastroesophageal junction cancer with disease progression on or after a trastuzumab-containing regimen (DESTINY-Gastric02): primary and updated analyses from a single-arm, phase 2 study. *The Lancet Oncology*. 2023;24(7):744-756. doi:<https://dx.doi.org/10.1016/S1470-2045(23)00215-2>

101. Nishikawa T, Hasegawa K, Matsumoto K, et al. Trastuzumab Deruxtecan for Human Epidermal Growth Factor Receptor 2-Expressing Advanced or Recurrent Uterine Carcinosarcoma (NCCH1615): The STATICE Trial. *Journal of clinical oncology : official journal of the American Society of Clinical Oncology*. 2023;41(15):2789-2799. doi:<https://dx.doi.org/10.1200/JCO.22.02558>

102. Perez-Garcia JM, Vaz Batista M, Cortez P, et al. Trastuzumab deruxtecan in patients with central nervous system involvement from HER2-positive breast cancer: The DEBBRAH trial. *Neuro-oncology*. 2023;25(1):157-166. doi:<https://dx.doi.org/10.1093/neuonc/noac144>

103. Shitara K, Yamaguchi K, Muro K, et al. Trastuzumab deruxtecan in patients with locally advanced or metastatic HER2-positive gastric cancer: a multicenter, open-label, expanded-access study. *International journal of clinical oncology*. 2024;29(1):27-35. doi:<https://dx.doi.org/10.1007/s10147-023-02422-x>

104. Daiichi Sankyo. Data from: DS-8201a in Patients With Cancer That Tests Positive for Human Epidermal Growth Factor Receptor 2 (HER2) Protein. 2020. *ClinicalTrials.gov*.

105. Denney WS, Duvvuri S, Buckeridge C. Simple, Automatic Noncompartmental Analysis: The PKNCA R Package. *Journal of Pharmacokinetics and Pharmacodynamics*. 2015;42(1):S65. doi:10.1007/s10928-015-9432-2
